# Incidental findings in and around the prostate on prostate MRI: a pictorial review

**DOI:** 10.1186/s13244-021-00979-7

**Published:** 2021-03-18

**Authors:** Janki Trivedi, Tom Sutherland, Mark Page

**Affiliations:** 1 Medical Imaging Department, St Vincent’s Health Melbourne, Ground Floor Main Hospital, 41 Victoria Parade, Fitzroy, VIC 3065 Australia; 2 Faculty of Medicine, University of Melbourne, Melbourne, VIC Australia

**Keywords:** Periprostatic, Incidental, Findings, MpMRI

## Abstract

Prostate MRI has seen rapid growth in use in recent years as an advanced diagnostic modality to detect focal areas of clinically significant prostate cancer, to identify an area for targeted biopsy and to guide management and surveillance. The increase in use has also led to increased diagnosis of incidental lesions arising from structures around the prostate. These incidental findings may be related to the genitourinary system or non- genitourinary system and may have a benign aetiology which needs no additional follow-up, or it may require surveillance and management. The field of view in a multiparametric prostate MRI includes other pelvic organs, neurovascular bundles, bowel, lymph nodes and bones. Being familiar with standard MRI characteristics and a sound knowledge of anatomy of the prostate and surrounding structures can help in distinguishing normal anatomy from pathology. Given that patients undertaking a prostate MRI are usually a cohort with increased anxiety from their known or suspicion of prostate cancer, it is important that radiologists are familiar with these common incidental findings to minimise anxiety to the patient, have a well-informed discussion with the referring clinician and reduce costs associated with unnecessary further testing and follow-up of benign incidental findings. Additionally, being able to diagnose more serious incidental pathologies early can be life-saving and potentially significantly alter patient management.

## Key points

- Familiarity with MRI signal characteristics of blood, calcifications and cyst fluid is important.
- Location of the incidental finding narrows the differential list as some pathologies occur more frequently in certain sites.
- Prostate abscess can have similar imaging characteristics to prostate cancer; history and clinical presentation is useful to distinguish.
- Recent prostate or peri-prostate interventions can mimic pathology.

## Introduction

Multiparametric MRI (mpMRI) prostate allows accurate anatomical and functional imaging of the prostate gland and diagnosis of significant (intermediate and high risk) prostate cancer [1]. mpMRI can also be utilised in active surveillance for patients with low- and intermediate-risk prostate cancer and guide targeted prostate biopsies.

Transrectal ultrasound (TRUS) and positron emission tomography (PET) are other imaging modalities which can be used to assess the prostate and aid in diagnosis and localisation, therapy, staging, active surveillance and recurrence monitoring [2]. On TRUS, a relatively safe and inexpensive imaging modality, prostate cancer is most often hypoechoic relative to the normal peripheral zone but may sometimes be isoechoic or even hyperechoic. Further features that increase confidence in diagnosing prostate cancer on TRUS are asymmetry in prostate size (particularly in the peripheral zone), capsular distortion and loss of differentiation between the central gland and peripheral zone [2].

mpMRI of the prostate typically combines the anatomical images of T1- and T2-weighted imaging with functional sequences including diffusion-weighted imaging (DWI) (with a calculated b value of 2000), in conjunction with the apparent diffusion coefficient (ADC), and dynamic contrast enhancement (DCE) T1 sequences (using gadolinium-based IV contrast agents) [1, 3]. The T2 sequence provides the best assessment of prostate margins for extracapsular extension, seminal vesicle invasion, neurovascular bundle and adjacent organ involvement [3]. T1 weighted imaging helps differentiate post-biopsy haemorrhage from tumour [3]. DWI is useful because prostate cancer has a reduced diffusion of water, compared with normal prostate, due to its tightly packed cells [1, 3]. DCE imaging provides further functional information as malignancy causes changes such as increased blood flow, neo-vascularity and leaky capillaries [1, 3]. Finally, magnetic resonance spectroscopy is a functional technique that indirectly measures metabolite levels of choline, creatinine and citrate in the prostate but, due to being technically challenging and time consuming, is often not included in a mpMRI protocol [1, 3].

The Prostate Imaging Reporting and Data system (PIRADS) is a structured reporting system allowing a weighted calculation on a 5-point scale and is based on the probability that a combination of the mpMRI parameters correlates with the presence of a clinically significant cancer [4]. An alternative method of reporting prostate MRI is using the five-point Likert scale where scores indicating higher suspicion (Likert 4–5) on MRI correlate strongly with a higher likelihood of overall cancer where a targeted biopsy might be useful [5].

In a prostate MRI, the field of view includes several key structures. Anterior relations of the prostate are pubic symphysis and the retropubic space of Retzius. Posterior to the prostate is the rectovesical fascia and rectum. The bladder is superior to the prostate, and the urogenital membrane lies inferior to the prostate. The posterior wall of the bladder contacts the seminal vesicles, ampulla of the vas deferens and the bladder venous plexus [6]. Seminal vesicles are located above and posterior to the prostate base. The prostatic neurovascular bundle (NVB) is situated laterally in the posterolateral angles of the prostate at 5 and 7 o’clock positions and gives off branches into the prostate at the apex and base [6].

For a radiologist reviewing or reporting a mpMRI prostate, it is crucial that they are familiar with the imaging findings related to prostate cancer, but they must also have an understanding of incidental findings in the field of view, which may be indolent or have a significant impact on the patient’s management.

Recent studies have found incidental findings in the range of 42% [7]–52.7% [8]. While the majority of true incidental findings were non-urological, 6.6% of these were considered clinically significant [7]. Incidental findings were noted to be more common in patients aged over 65, with a ratio of 57% versus 46% in patients aged under 65 [8]. 4.2% of patients required surgery for incidental findings including bladder cancer (1.1%), testicular tumour (0.5%) and rectal cancer (0.3%) [8], further affirming the importance of detection and interpretation of incidental findings early.

The purpose of this pictorial review is to illustrate some common incidental findings, as classified by systems and outlined in Table 1, that the radiologist may encounter when reporting a prostate MRI. These incidental findings can have clinically significant as well as indolent outcomes, and early and accurate identification can alter management. Being familiar with the anatomy of the prostate and peri-prostatic spaces and pertinent MR signal characteristics can help formulate differential diagnoses for an incidental finding while the patient’s age, clinical history and recent interventions, if any, can help further narrow the differential list.

**Table 1:** Commonly seen incidental findings on prostate MRI classified by systems

Space	Benign	Malignant
Prostate and periprostatic region	Seminal vesicle stones, prostate calcifications, utricle cyst*, Mullerian duct cyst*, Cowper’s gland duct cyst, periprostatic haematoma, prostate abscess, periprostatic dermoid *May rarely develop malignant degeneration	Prostate cancer with extraprostatic extension, undifferentiated pleomorphic sarcoma (UPS) of the spermatic cord, spermatic cord lymphoma
Musculoskeletal	Tumour: intramuscular lipoma, periprostatic leiomyoma and solitary fibrous tumour Non-tumour: degenerative disc disease, avascular necrosis, trochanteric bursitis, tendon ruptures	Skeletal metastasis, soft tissue sarcoma (liposarcoma, leiomyosarcoma, UPS), parachordoma
Urinary	Bladder diverticula, bladder calculi	Urothelial carcinoma
Colorectal	Polyps, haemorrhoids, diverticulitis, inguinal hernia, ascites* *May be due to benign or more sinister cause	Rectal adenocarcinoma, rectal villous adenoma, rectal GIST
Neurovascular	Periprostatic venous varix, aneurysms	Nerve sheath tumours, Lymphadenopathy* *may be reactive, infective/ inflammatory or malignant

*Indicates that those two conditions of utricle cyst and mullerian duct cyst may rarely develop malignant degeneration

Our case selection depicts the spectrum of cases encountered in daily practice in a tertiary referral centre over the past five years in patients’ having prostate MRI which is influenced by the demographics and history of the patients referred to our department. We highlight some cases of incidental findings that can be seen in spaces and structures around the prostate classified by systems in Table 1, as well as within the prostate and periprostatic region which are further outlined in Table 2.

**Table 2:** Lesions arising from the prostate or periprostatic region

Periprostatic mass	Benign	Malignant
Solid	Tumour: lipoma, leiomyoma and solitary fibrous tumour Non-tumour: Haematoma, Dermoid*	Soft tissue sarcoma (liposarcoma, leiomyosarcoma, UPS) Nerve sheath tumours GIST
Cystic	Mullerian duct cyst*, Utricle cyst*, Cowper’s gland duct cyst, seminal vesicle cysts	Cystic nerve sheath tumour

*May rarely develop malignant degeneration

## Prostate and periprostatic region

### Haematospermia with and without stone in seminal vesicles

On T2W images, seminal vesicles appear as high-intensity sac-like structures and can be easily differentiated from significantly lower signal intensity of periprostatic fat. On T1W images, this signal pattern is reversed, and seminal vesicles have lower signal intensity compared to periprostatic fat [6].

Haematospermia is the presence of blood in the seminal fluid. Normal appearances of the seminal vesicles are grape-like clusters with high T2 signal intensity of internal content and low T2 signal intensity of the wall. The size of normal seminal vesicles is highly variable. Given blood has intrinsic high T1 signal; this is on its own a benign and usually straightforward diagnosis to make. However, high T1 signal can also be seen in the seminal vesicles with no haematospermia due to proteinaceous content. Haematospermia is usually a benign and self-limiting condition commonly seen in young sexually active males but albeit invokes anxiety in the patient and referrer.

**Fig. 1:**
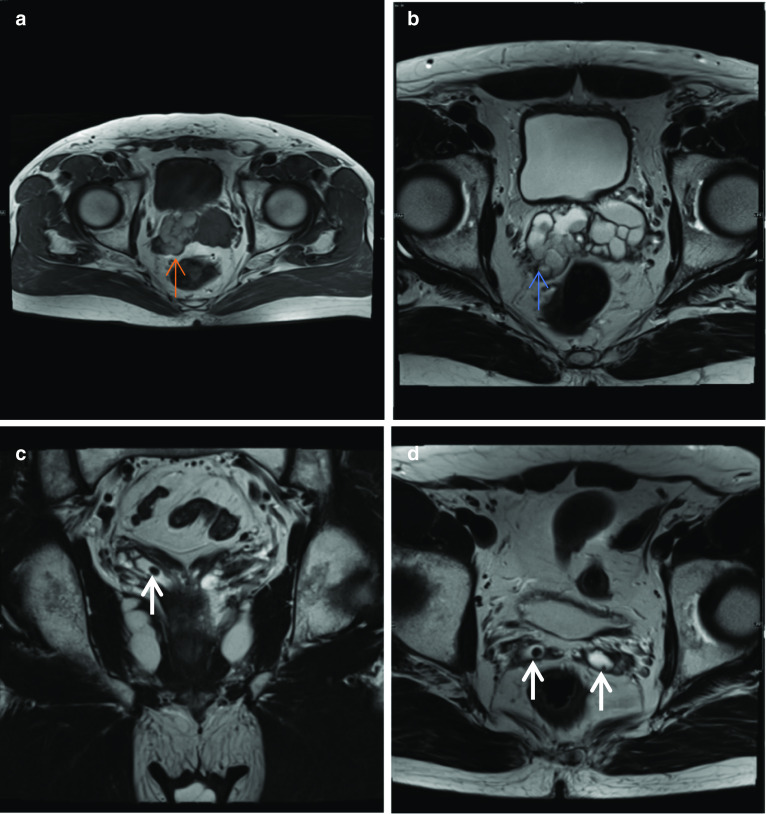
*Haematospermia* (**a**) Axial T1W image shows intrinsic T1 hyperintensity within the right seminal vesicle (red arrow). **b** Axial T2W image shows corresponding T2 hypointensity in this region in keeping with blood products (blue arrow). **c** Coronal T2W and (**d**) axial T2W images in a different patient show rounded low T2 signal intensity lesions, in the right seminal vesicle in (**c**) and bilaterally in (**d**) but larger in the right seminal vesicle, in keeping with stones (white arrows)

In Fig. 1a–d, the cause for haematospermia is also demonstrated, which is a small stone in bilateral seminal vesicles. Calculi are low T2 signal on MRI. Seminal vesicle stones are rare and are postulated to be associated with urinary tract infections, anomalies, obstruction and reflux into the ejaculatory duct [9]. Other aetiologies include prior biopsy or focal therapies; however, often the cause remains idiopathic. Patients presenting with stones in the seminal vesicles may be asymptomatic or symptomatic with haematospermia, testicular pain, painful ejaculation, low ejaculate volumes or infertility [9].

### Prostate calcifications

**Fig. 2:**
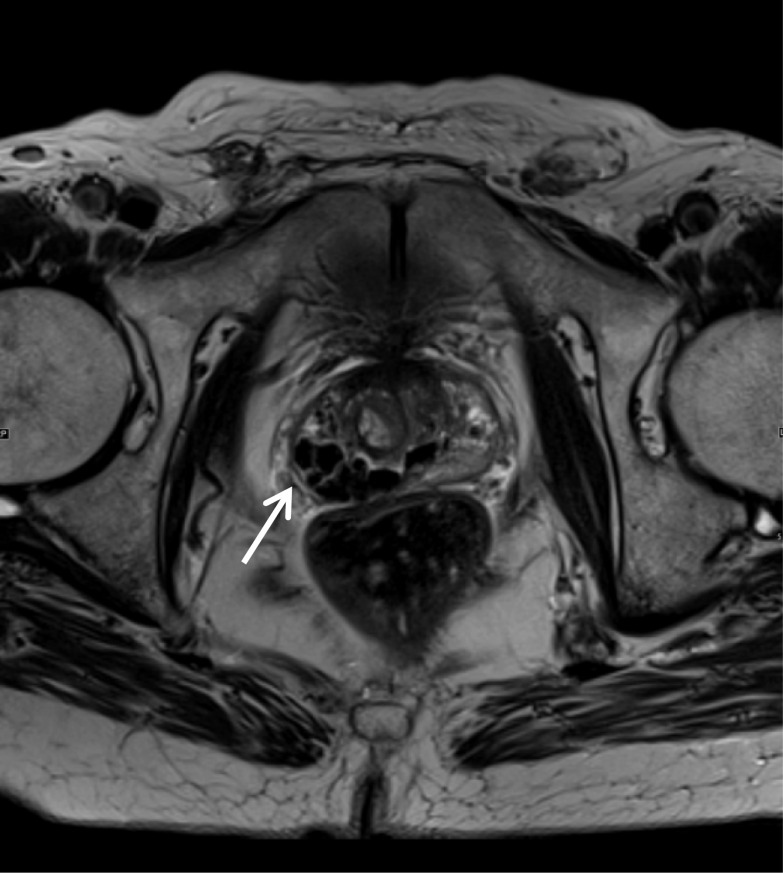
*Prostate calcification* Axial T2W image shows well-defined low T2 signal lesions in the right posterolateral prostate in keeping with prostate calcification (white arrow)

Calcifications typically have intrinsic low T2 signal and T1 signal. Figure 2 demonstrates a case of prostate calcifications. Prostate calcifications are non-specific and in the young population may be associated with prostatitis, infection or inflammation while benign prostatic hyperplasia (BPH) is the most common cause in the elderly [10]. Most calcifications are found within the transition zone, and they are less frequently seen in the peripheral zone.

### Prostatic utricle cyst

**Fig. 3:**
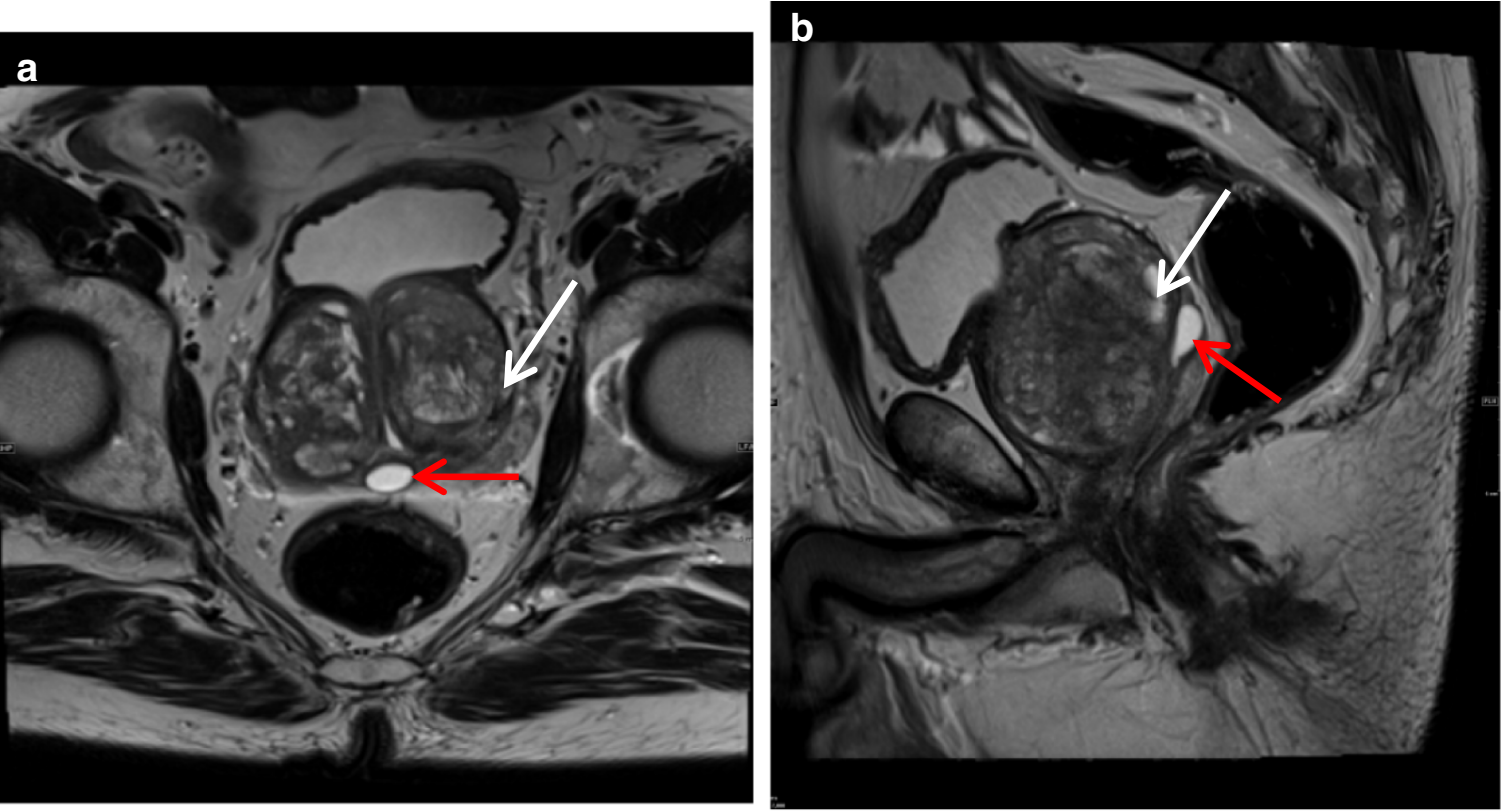
*Prostatic utricle cyst* (**a**) Axial T2W image shows a high T2 signal pear-shaped cystic lesion (red arrow) in the midline posterior to the prostate (white arrow), and on the sagittal image (**b**), it can be seen that it does not extend above the base of the prostate in keeping with a utricle cyst

A prostatic utricle cyst is an area of focal dilatation that occurs within the prostatic utricle. It always arises from the level of the verumontanum and is always in the midline. Utricle cysts do not extend above the level of the prostate base, while Mullerian duct cysts, which are discussed in detail further in the article, may. In contrast, a Mullerian duct cyst can arise anywhere along the path of Mullerian duct regression from scrotum to utricle [11]. Prostatic utricle cysts tend to be smaller (less than 10 mm) in size. While prostatic utricle cysts can be associated with genitourinary abnormalities such as hypospadias, cryptorchidism and unilateral renal agenesis; Mullerian duct cysts don’t have such associations [11]. Urine may pool in utricle cysts since they communicate with the urethra, whereas mullerian duct cysts do not communicate with the urethra. Figure 3 demonstrates a small utricle cyst (red arrow). In our experience, these cysts are a common incidental finding.

### Mullerian duct cyst

**Fig. 4:**
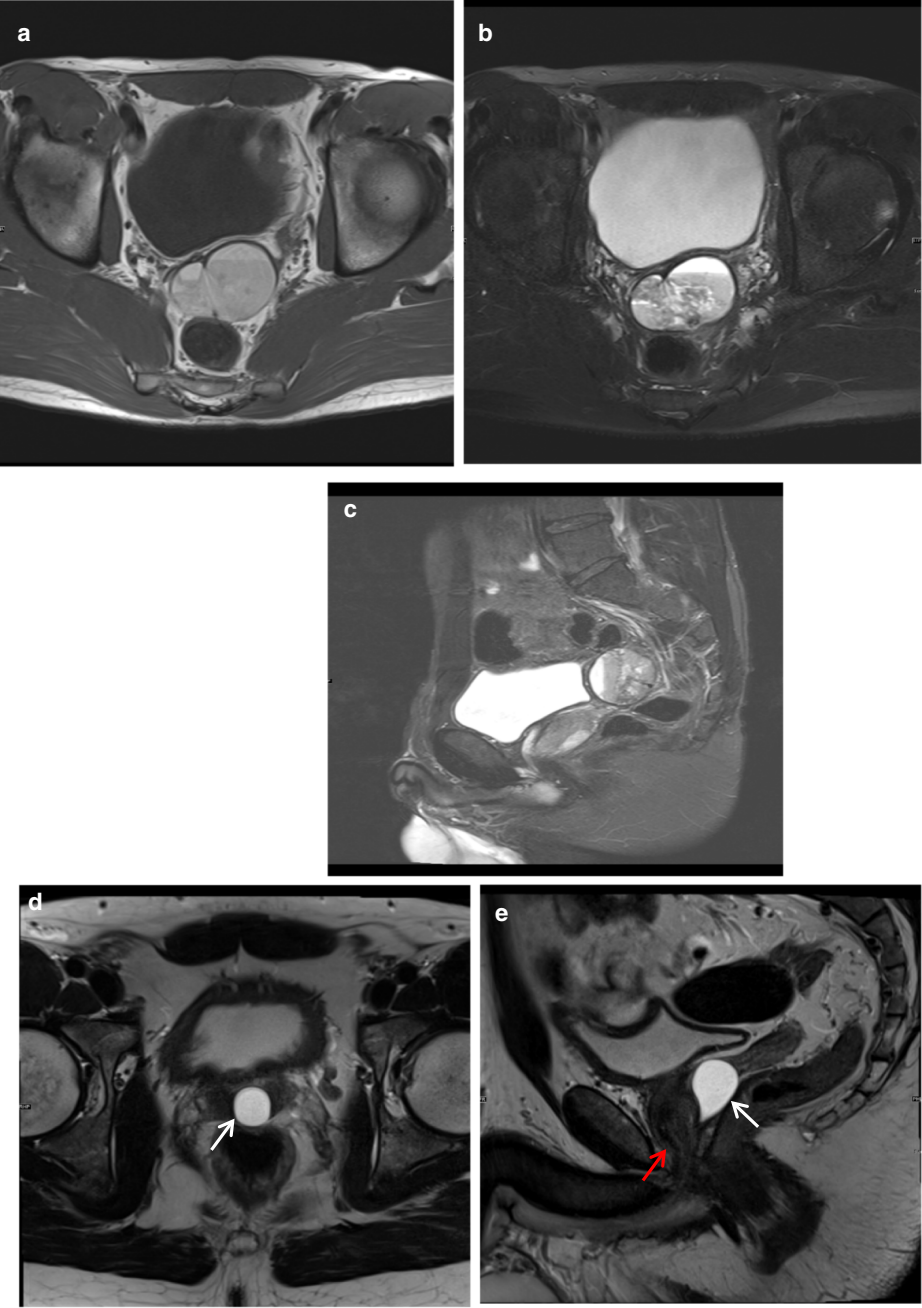
*Mullerian duct cysts* Axial **a** T1W and (**b**) T2W images and Sagittal (**c**) STIR images show a cystic structure with a fluid–fluid level from high signal intensity simple fluid and low signal intensity fluid from haemorrhage. It extends beyond the base of prostate. **d** Axial and (**e**) sagittal T2W images show another case with a midline cystic structure (white arrows) with homogeneous high T2 signal which is bulbous at the superior aspect with an inverted droplet morphology inferiorly. The base of the lesion does extend above the level of the base of the prostate gland which is shown by the red arrow. These are in keeping with Mullerian duct cysts

Müllerian duct cysts are mesodermal in origin with peak incidence in men 20–40 years of age, with a reported prevalence of less than 1%. The clinical manifestation mainly depends on the size of the cyst, concurrent infections and secondary lesions or deformity [12]. They may be asymptomatic or can manifest with urinary retention, urinary tract infection or ejaculatory impairment due to ejaculatory duct obstruction. Additionally, patients may experience a discomfort in the perineum and can present with persistent haematospermia or infertility caused by obstructive azoospermia [13]. Müllerian duct cysts appear as typical teardrop-shaped or oblong midline cysts extending above the posterior superior margin of the prostate and are best seen in the sagittal plane [12]. They do not communicate with the posterior urethra and do not contain spermatozoa. At MR imaging, Müllerian duct cysts show fluid signal intensity at T1- and T2-weighted MR imaging but may show high T1 and T2 signal intensity due to mucinous material, haemorrhage or infection [11]. The treatment of Mullerian duct cysts is usually determined based on the associated clinical symptoms and complications and can range from needing no treatment or surveillance to intervention in the form of transrectal ultrasound-guided aspiration and injection of sclerosing agents. Surgical treatments involve transurethral cyst incision drainage, open cyst resection and laparoscopic cystectomy [13] (Fig. 4).

### Cowper’s gland duct cyst

**Fig. 5:**
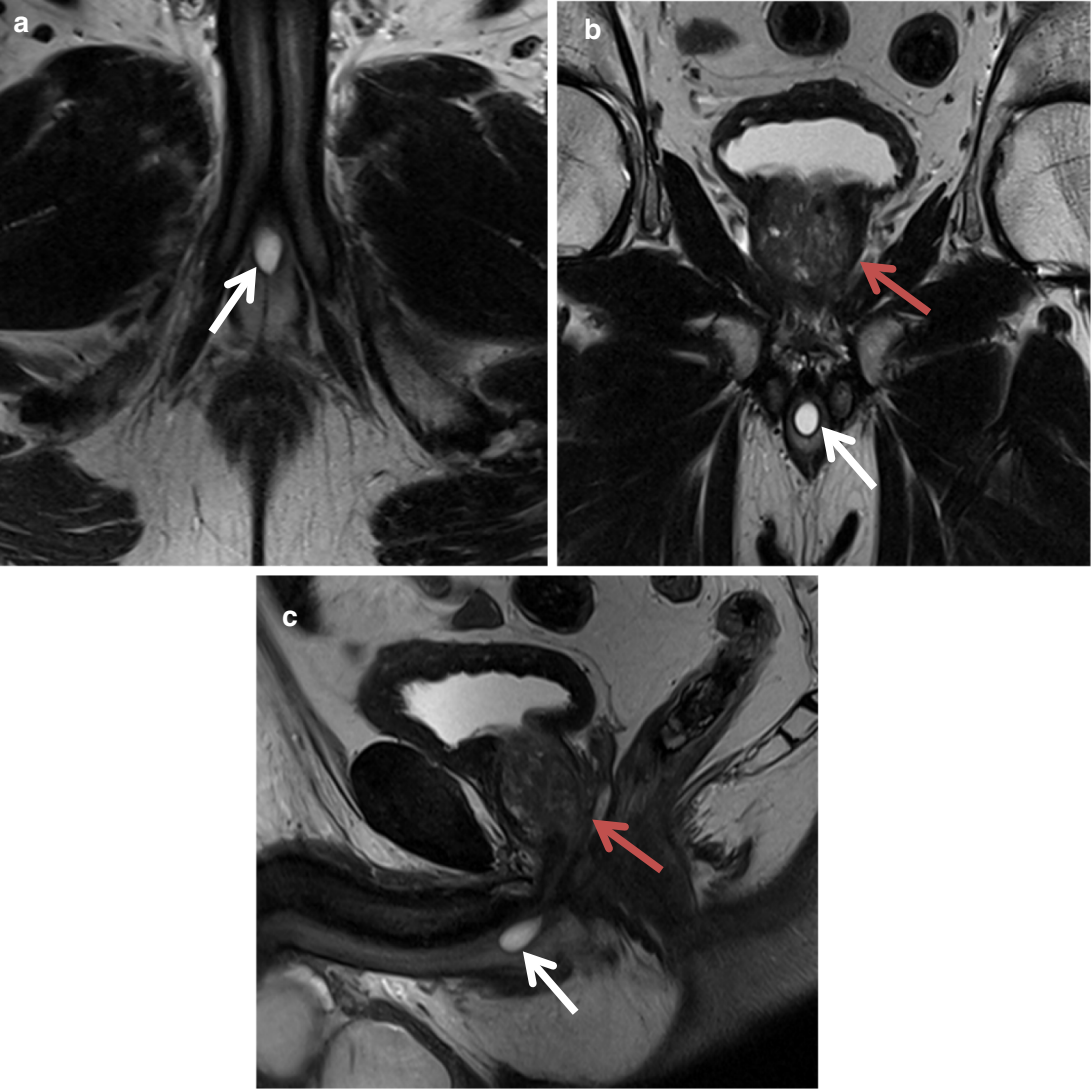
*Cowper’s gland duct cyst* **a** Axial, (**b**) coronal and (**c**) sagittal T2 weighted images show a midline ovoid high T2 signal lesion at the penile base (white arrow) in keeping with a Cowper’s gland duct cyst. The prostate is seen superior to the lesion (red arrow)

Cowper’s glands, or bulbourethral glands, are paired pea-sized glands of the male reproductive tract. They are located in the deep perineal pouch posterolateral to the membranous portion of the urethra and covered by the external urethral sphincter [14]. Each gland secretes mucus through individual ducts, 2.5 cm in length, which descend posterolateral and parallel to the membranous urethra through the perineal membrane and corpus spongiosum of the bulb of the penis to enter the floor of the bulbar urethra [14]. They produce fluid that has a role in urethral lubrication and sperm motility during ejaculation. Cysts in the Cowper’s gland duct can cause varying symptoms depending on their size and location however common symptoms include diminished urinary stream, urinary retention, frequency and bloody urethral discharge. The main differential diagnoses are incomplete urethral duplication and inflammatory or traumatic diverticula [15]. On MRI, they are seen in the midline as an ovoid cystic structure at the penile base adjacent to the ventral aspect of the proximal bulbous urethra. They demonstrate classic features of a cyst with low T1 and high T2 signal intensities. Endoscopic resection of the septum between urethra and Cowper’s duct cyst is the treatment of choice providing relief of voiding symptoms [15] (Fig. 5).

### Periprostatic haematoma post-biopsy

Haematoma around the prostate gland is a rare complication following transrectal ultrasound-guided biopsies but if large it can lead to serious haemorrhage and hypovolaemia in some patients.

**Fig. 6:**
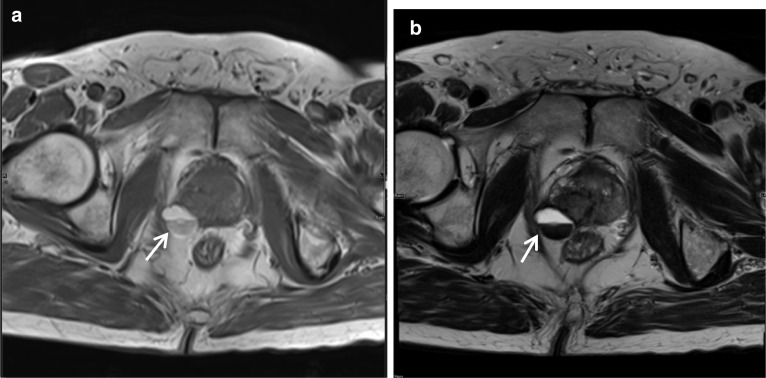
*Periprostatic haematoma post-biopsy* **a** Axial T1W and (**b**) T2W images demonstrate a right posterolateral periprostatic haematoma (white arrow) post-biopsy which is high signal on both sequences with a fluid–fluid level

Performing an MRI post a prostate biopsy in the acute or subacute period is not routine, and hence, they are a useful entity for the radiologist to be familiar with. Additionally, the damage induced by the biopsy needle during the procedure may cause a transient irregularity of the periprostatic capsule which may lead to an overestimation of extraprostatic tumour extension [16]. As with haematoma elsewhere, in this setting too on MRI it is high signal on T1- and T2-weighted sequences. Clinical history regarding recent intervention is crucial to establishing this diagnosis with confidence. If small, they are usually self-limiting but may be followed-up to ensure resolution and conservative management with simple analgesia is often adequate. Table 3 outlines a summary of common periprostatic lesions and their imaging findings (Fig. 6).

**Table 3:** Summary of common periprostatic lesions and characteristic MR findings

Periprostatic lesion	MR findings
Prostatic utricle cyst	High T2, low T1, always in the midline, communicate with urethra
Mullerian duct cyst	High T2, low T1, usually extend above the prostate, no communication with urethra
Cowper’s duct cyst	High T2, low T1, at penile base adjacent to ventral bulbous urethra
Periprostatic haematoma post-biopsy	High T1 and high T2 signal, high T1 signal helps differentiate tumour from post-biopsy haemorrhage, may show a fluid–fluid level
Seminal vesicle cyst	High T2, low T1, may show high T1 signal if associated with haemorrhage
Prostatic retention cyst	High T2, low T1, smooth-walled unilocular cysts, may occur in any glandular zone of the prostate

### Prostate TB abscess in a patient with bladder TCC after intravesical instillation of BCG

Adjuvant intra-vesical immunotherapy with BCG for bladder transitional cell carcinoma can lead to a rare complication of tuberculous infection in the prostate via haematogenous spread and direct extension which can cause a prostate abscess [17].

**Fig. 7:**
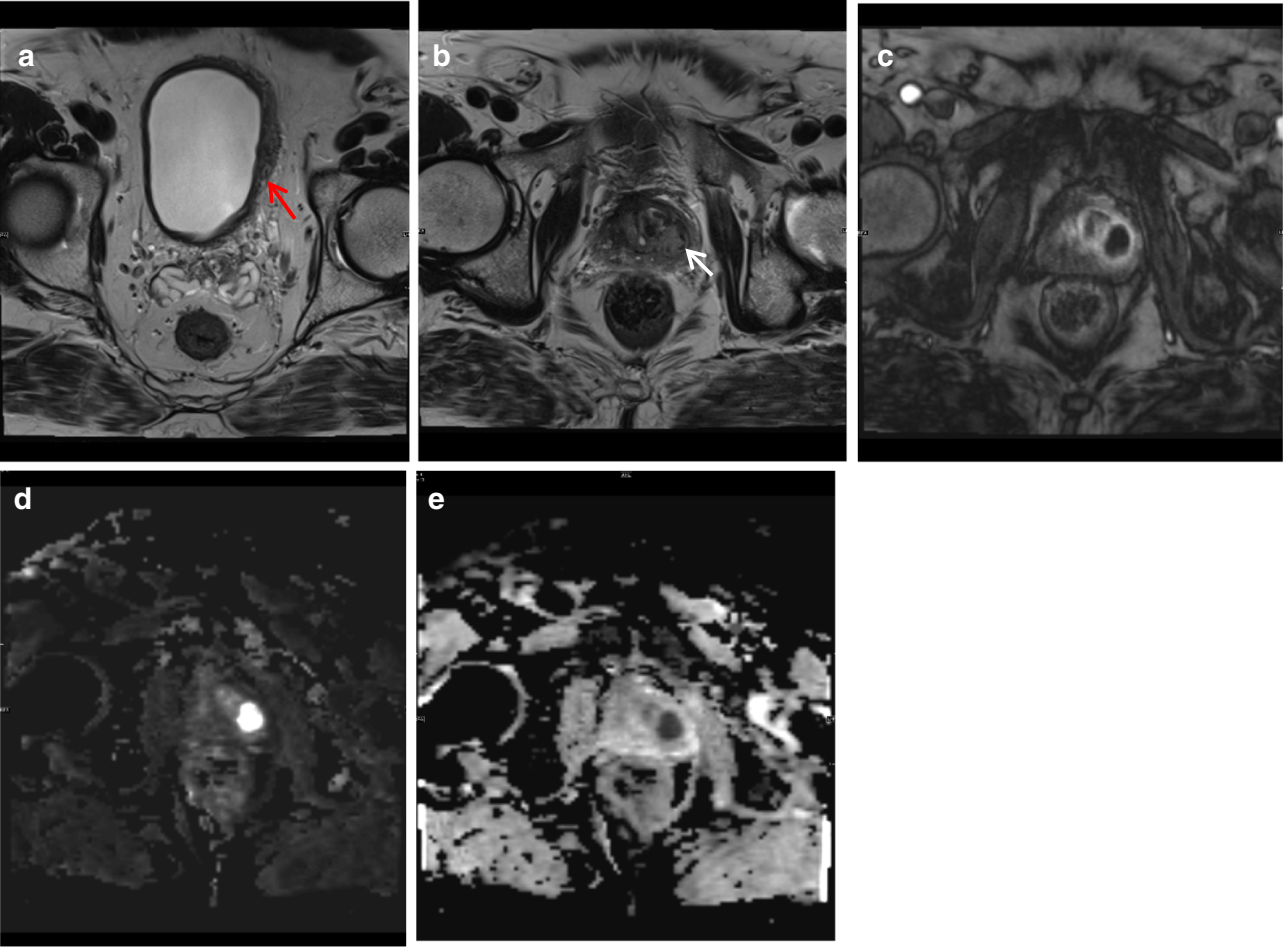
*Prostate abscess* **a**, **b** Axial T2W images show left lateral bladder wall thickening (red arrow) and heterogeneous but predominantly low T2 signal in the left prostate (white arrow), respectively. **c** Post-contrast, the lesion demonstrates strong peripheral contrast enhancement and on DWI/ ADC in **d**, **e**, respectively, the central element of the left prostate lesion shows restricted diffusion with a very low ADC value

The granulomatous reaction that occurs in prostate tissues following the topical instillations of BCG after resection of bladder cancer can present MRI features similar to prostate cancer [16]. It appears as an ill-defined region with moderate contrast enhancement, diffusion restriction and very low ADC values due to the high cellular density. These are a well-recognised cause of false positive MRI. However, frank abscess formation related to BCG is rare with only a handful of cases described in the literature. History and biochemical correlation are important to consider this as a differential diagnosis and a biopsy may be needed to confirm (Fig. 7).

### Periprostatic dermoid cyst

**Fig. 8:**
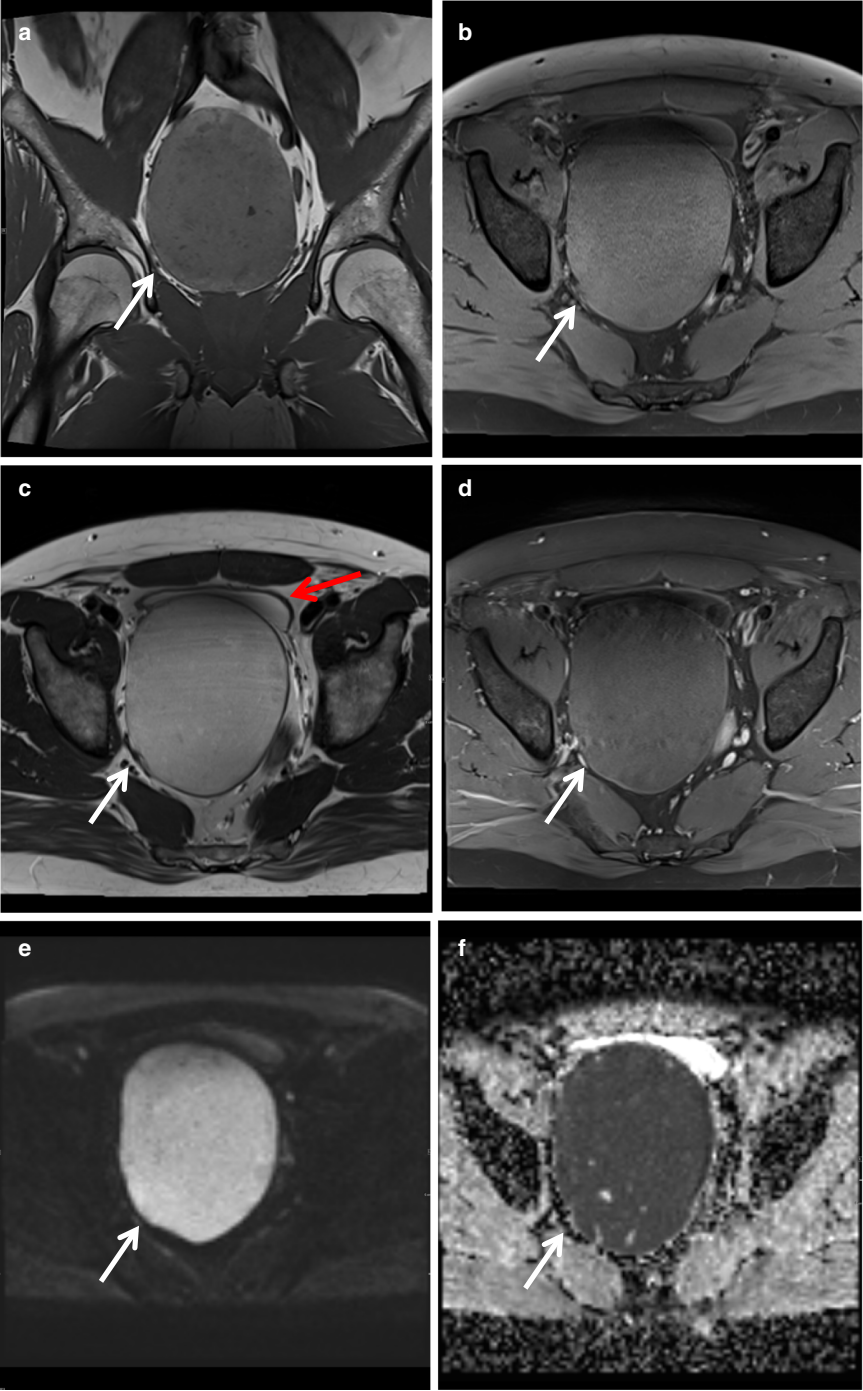
*Periprostatic dermoid cyst* **a** Coronal T1W image shows a large well-circumscribed mass in the pelvis (white arrows) which is mildly T1 hyperintense to muscle, shows mild fat saturation on (**b**) axial T1 fat saturation sequence. **c** Axial T2W image shows the lesion is high T2 signal compared to muscle. It causes significant mass effect on the bladder (red arrow) displacing it anteriorly and exerts mass effect on the prostate and seminal vesicles. **d** Post-contrast T1 shows no significant enhancement. The lesion shows strong diffusion restriction with (**e**) high DWI and (**f**) low ADC. No evidence of calcification, fluid–fluid level or suspicious enhancing nodular soft tissue thickening was identified

Dermoid cysts or mature teratomas are generally benign tumours which originate from uncontrolled proliferation of pluripotent or germ cells and embryonal cells [18]. While teratomas of germ cells are found in testes and ovaries, teratomas of embryonic cells sources are always congenital and found in extragonadal locations. Teratomas of the retroperitoneum are rare in males, are usually asymptomatic and discovered incidentally. However, depending on their size and location, they may exert mass effect on surrounding abdominal or pelvic organs causing symptoms depending on the organ they are displacing. If large, urinary symptoms such as frequency and retention, abdominal pain and increased bowel movements are commonly reported [18]. Physical examination may reveal an abdominal or pelvic mass and distension. On MRI, the features of a dermoid cyst are variable and depending on their content they may be solid, cystic or mixed. Presence of calcification, fluid–fluid levels, fat and enhancing components is also variable. Pathology usually shows a cystic mass lined by mature squamous epithelium with skin adnexae and hair shafts with keratin [18]. Surgical resection is the mainstay of treatment and allows symptomatic relief and definitive histological diagnosis (Fig. 8).

### Prostate adenocarcinoma with a large right seminal vesicular cyst

**Fig. 9:**
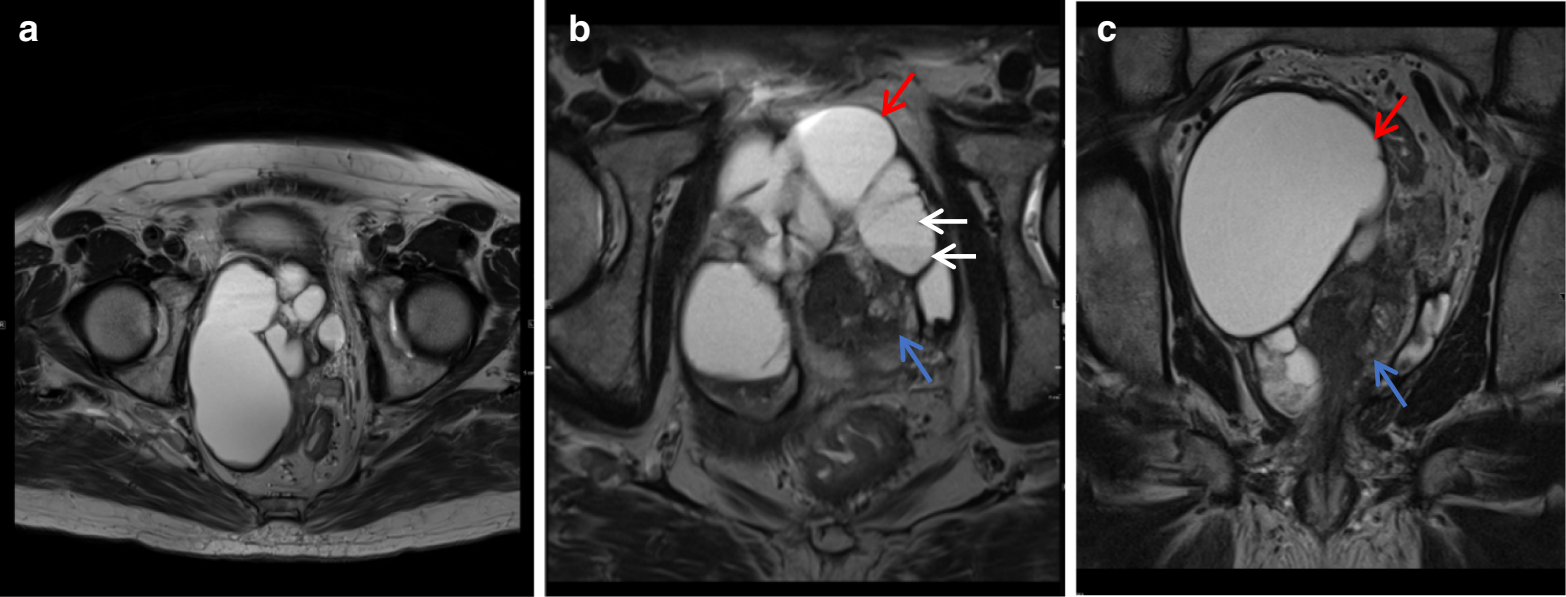
*Cystic prostate adenocarcinoma* **a**–**c** Axial T2W images demonstrate multiple large high T2 signal lesions in keeping with cysts (red arrow) surrounding the prostate (blue arrows) with small fluid-fluid levels (white arrows) likely due to internal haemorrhage

Malignancy associated with cystic lesions of the prostate is rare. Both benign and malignant prostate neoplasms may contain cystic components. In this case, histology post-radical prostatectomy revealed Gleason score 5 + 4 = 9 prostate adenocarcinoma with involvement of both seminal vesicles. The large right-sided cystic structure was identified as arising from the right seminal vesicle which was invaded by the adenocarcinoma, leading to obstruction and distention. Due to the cystic nature of the lesion, there was no avidity within it on PSMA PET. When the cystic component grows as large as in this case, it can be associated with lower urinary tract symptoms. Other tumours of the prostate gland which can have cystic components include papillary cystadenocarcinoma and combined transitional cell/ adenocarcinoma and leiomyoma or liposarcoma can also show this although are much rarer [19]. The aspirate of the cystic component is usually haemorrhagic and contains malignant cells with a high concentration of prostate specific antigen and Y-seminoprotein [19] (Fig. 9).

## Musculoskeletal

### Periprostatic leiomyoma

**Fig. 10:**
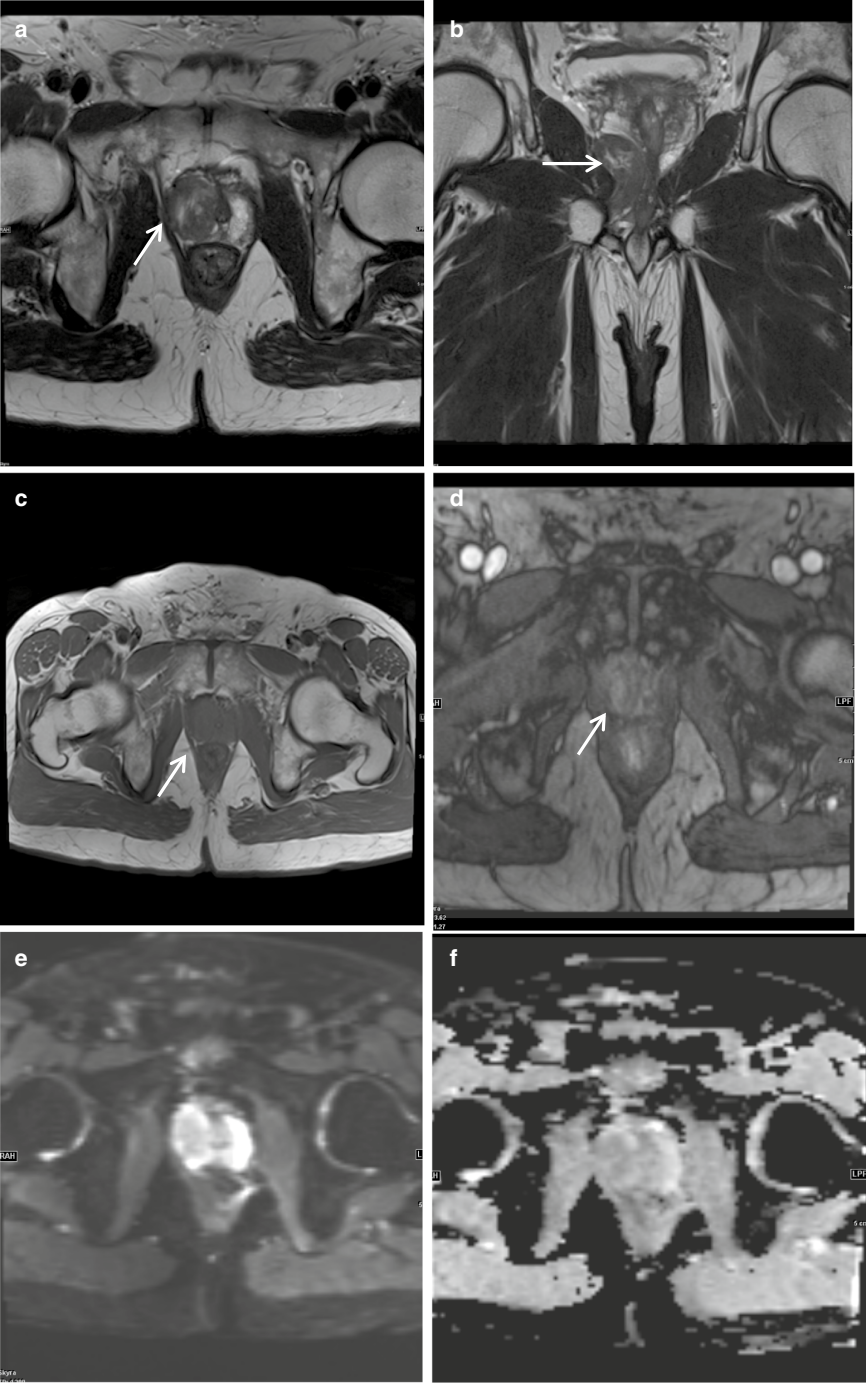
*Periprostatic leiomyoma* **a** Axial and (**b**) Coronal T2W images demonstrate a right periprostatic mass (white arrow) which indents the right apex from below displacing it superiorly and to the left with mild distortion of the prostatic urethra. Relative to muscle, it is slightly T2 hyperintense with some intrinsic high T2 signal foci. **c** Axial T1W image shows the lesion is isointense to prostate and in (**d**) the lesion shows mild heterogeneous contrast enhancement. On DWI/ ADC, **e**, **f** respectively, the lesion showed some diffusion restriction

A leiomyoma occurs in all organs containing smooth muscle cells but most frequently in the gastrointestinal and female urogenital tract. Leiomyoma of the prostate gland or urethra is an extremely rare benign tumour which on histology and immunohistochemistry shows spindle smooth muscle cells without glandular elements, expressing smooth muscle actin (SMA) and desmin [20]. Leiomyoma of the prostate lesions tend to be normocellular with no or rare mitotic activity and no or rare nuclear atypia [20]. This is in contrast to leiomyosarcoma of the prostate which tends to show hypercellularity, evidence of infiltration, variable atypia, definite mitotic activity and necrosis [21]. On MRI, leiomyoma tends to be a well-defined non-invasive mass originating from the prostate. It can be homogeneous or contain areas of cystic degeneration. The non-cystic regions are T1-isointense and T2-hypo to slightly hyperintense relative to muscle on MRI [20]. Diffusion restricted imaging often shows diffusion restriction with low ADC values although in our cases did not diffusion restrict. DCE does not significantly help in establishing the diagnosis and it can have enhancement with a non-specific curve pattern [20]. Pelvic leiomyomas are rare in males and their incidence has been described as 0.5–1.2% only, among all retroperitoneal tumours [20]. Differentials include leiomyosarcoma, stromal tumour of unknown malignant potential (STUMP) or fibrous tumours for larger lesions while a small leiomyoma can be difficult to differentiate from a stromal hyperplastic nodule on imaging only [20]. In this case, the configuration of the mass conforming to the borders of the prostate apex and right levator sling with lack of invasion of these structures despite the large size favour a benign lesion. While asymptomatic patients can be managed conservatively with active surveillance, surgical treatment options include radical prostatectomy, transurethral resection or prostate artery embolisation [20] (Fig. 10).

### Periprostatic leiomyosarcoma

**Fig. 11:**
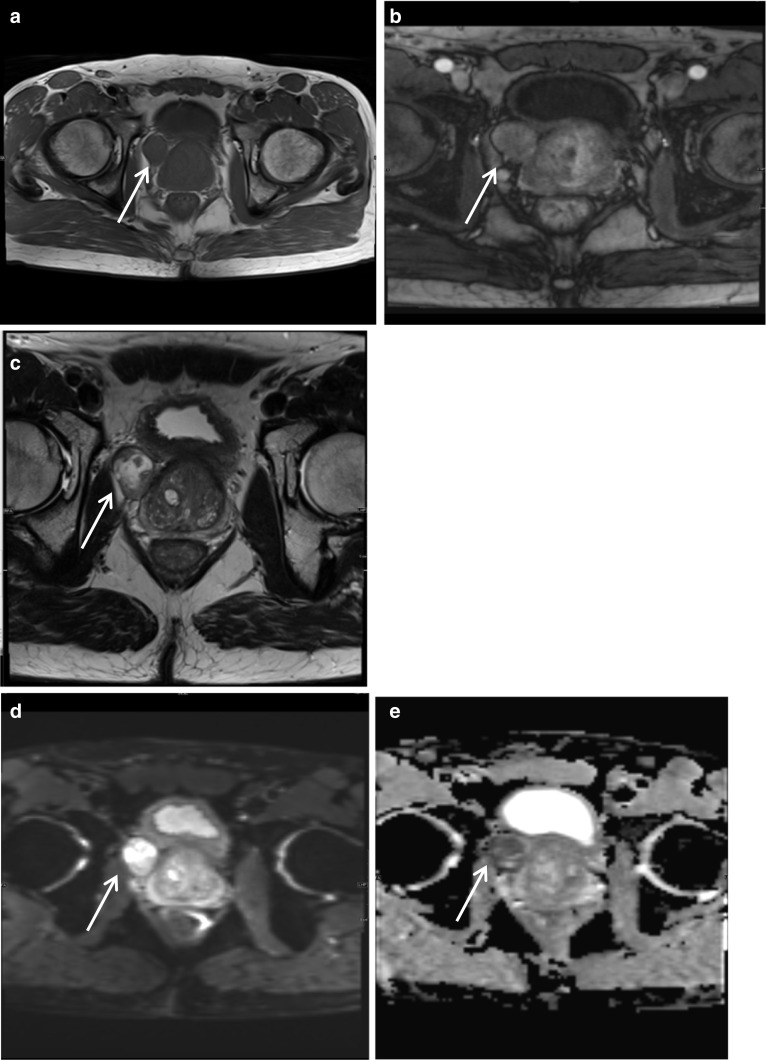
*Periprostatic leiomyosarcoma* **a**, **b** Axial T1W pre- and post-contrast images, respectively, show a rounded lesion arising from the right anterolateral prostate (white arrow) which is T1 isointense to prostate and shows uniform contrast enhancement. **c** Is a T2W image showing the lesion is predominantly T2 hyperintense lesion with no convincing invasion of the prostatic capsule. On (**d**) DWI and (**e**) ADC, respectively, the lesion shows restricted diffusion

Sarcomas of the prostate gland are rare, accounting for 0.1–0.2% of primary prostate cancers. Out of these, leiomyosarcomas are the most common in adults and account for approximately 25% of all sarcomas of the prostate gland [22]. Primary leiomyosarcoma of the prostate is rare and very aggressive and most commonly diagnosed in the sixth decade [22]. Patients may present with lower urinary tract symptoms, haematuria and perineal pain. On examination, a palpable hard mass can be found in the periprostatic region. Diagnosis is made on histology and immunohistochemistry which show spindle-shaped cancer cells and tend to be positive for vimentin, CD 44, smooth muscle actin and calponin, focally positive for desmin and at times positive for keratin [22]. Differentials include hyperprostatic stroma or leiomyoma. On MRI, they appear as solid lesions with variable intensity on T2-weighted images with enhancement of the solid components post-contrast administration [22]. This lesion showed similar imaging characteristics to the transition zone on all sequences except for having low ADC values and elevated DWI signal intensity. Surgery, potentially with adjuvant chemotherapy, remains the mainstay of treatment for leiomyosarcomas of the prostate that are operable; however, prognosis is poor and most cases are diagnosed in an advanced stage of the disease. They have a median survival of 3 to 4 years with a high risk of recurrence and metastasis to the liver and lungs [22] (Fig. 11).

### Solitary fibrous tumour

**Fig. 12:**
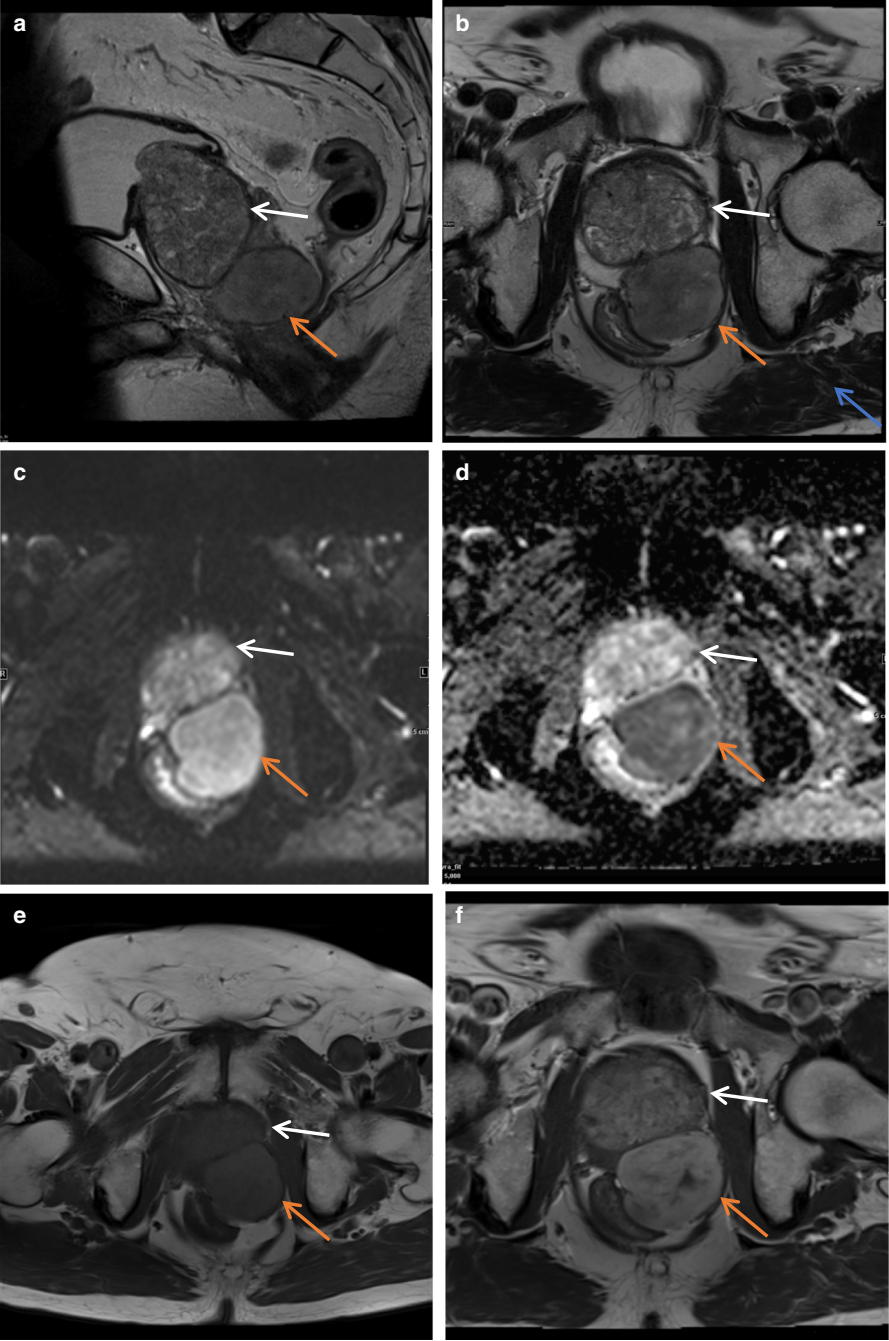
*Solitary fibrous tumour* A well-circumscribed homogeneous lesion (red arrow) arising from the left posterolateral prostate wall is seen in (**a**) sagittal and (**b**) axial T2W images as isointense to prostate (white arrow) and hyperintense to gluteus muscle (blue arrow). **c** DWI and (**d**) ADC images demonstrate diffusion restriction in the lesion. In **e** and **f** the lesion is iso- to mildly T1 hyperintense to prostate and shows uniform contrast enhancement. This was biopsy-proven solitary fibrous tumour

Solitary fibrous tumour is a rare spindle cell neoplasm which is most commonly seen in the pleura and thought to arise from mesenchymal fibroblastic cells [23]. It is exceedingly rare to occur within the prostatic region and should be differentiated from other spindle cell tumours of the prostate such as sarcoma and fibrosarcoma, usually requiring biopsy to establish a diagnosis. It must also be distinguished from hyperplastic prostatic stroma which can sometimes form a discrete nodule. SFTs most commonly present in the fifth and sixth decades of life with no significant gender predilection. SFT are often incidentally found on imaging. At MRI, they are usually isointense to muscle on T1-weighted images and variable on T2-weighted images, with what has been described as a black-and-white-mixed pattern [23]. Rounded or linear low-intensity foci may be seen on both T1- and T2-weighted images which is due to the collagen content, low cellularity and associated reduced proton mobility [23]. SFTs are vascular tumours which demonstrate vivid post-contrast enhancement [23]. This combination of features has been likened to a chocolate chip cookie appearance which can help in diagnosis. On 18F-FDG PET, benign SFT exhibit low-grade activity while malignant SFT tends to be hypermetabolic. Multiple lesions also raise the likelihood of malignant SFT. Overall, approximately 15–20% of SFT are malignant, and even benign SFT have indeterminate malignant potential. Therefore, complete resection is the treatment of choice [23] (Fig. 12).

### Lipoma and liposarcoma

Lipomas are common benign tumours composed of mature adipocytes. As in this case, a small amount of non-adipose tissue may be present and may represent focal fat necrosis, traversing blood vessels or muscle fibres. The non-adipose components must be assessed to exclude any aggressive features. Intramuscular lipomas are often lobular. They are straightforward to diagnose because of consistent adherence to subcutaneous fat signal on all sequences. They are high signal on T1-weighted sequences and show no to minimal enhancement. They show a high T2 signal on fast spin echo T2 and saturate on fat-saturated sequences [24]. Whereas liposarcomas can be large, septated, also with high T1 signal but demonstrate heterogeneous post-contrast enhancement. Other differentiating features more suggestive of a liposarcoma are nodules, thick soft tissue strands and diffusion restriction [24].

According to WHO classification, well-differentiated liposarcoma are generally called atypical lipomatous tumour (ALT) when located in the extremities or in the trunk to differentiate them from their intrathoracic or intraabdominal counterparts, which are more difficult to completely excise [25].

ALTs are generally resected with wide margins as they have a high rate of local recurrence and potential for dedifferentiation into high-grade sarcomas and metastatic spread.

It can be very difficult to differentiate a lipoma from and ALT on imaging. A recent publication comparing the two on MRI found that larger size, proximal lower limb location, deep to superficial fascia, incomplete fat suppression and increased architectural complexity were independent predictors of ALT when compared to benign lipomas [25].

**Fig. 13:**
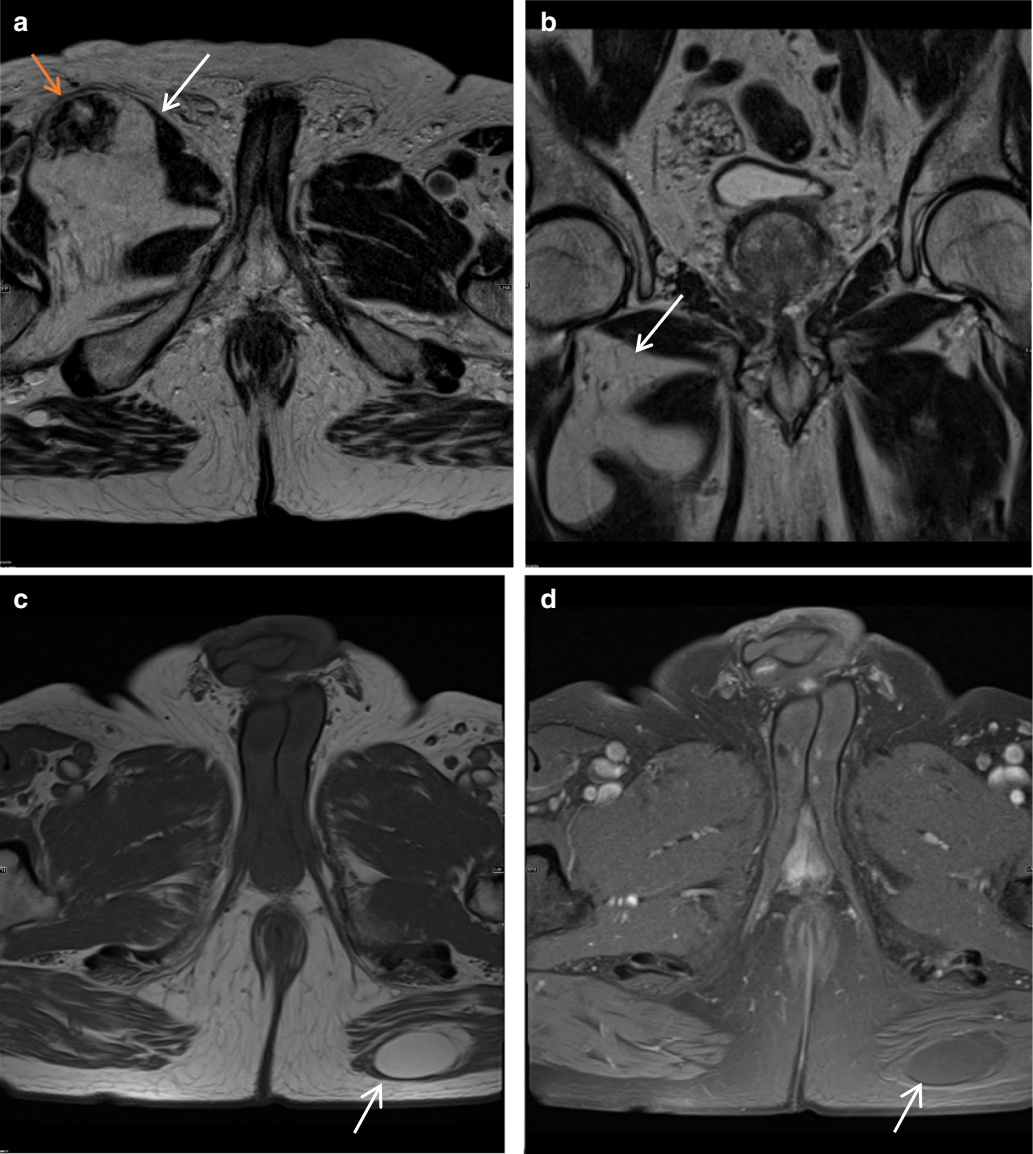
*Lipoma* **a** Axial and **b** coronal T2W images demonstrate a lipomatous mass within the right adductor compartment (white arrows) which is high T2 signal with a heterogeneous solid component at the anterior aspect (red arrow) which on biopsy represented a region of fat necrosis. **c** Axial T1 and (**d**) axial T1 fat sat post-contrast sequences show an ovoid lesion in the left gluteus maximus which is high T1 signal and suppresses on the fat sat sequence in keeping with a lipoma

**Fig. 14:**
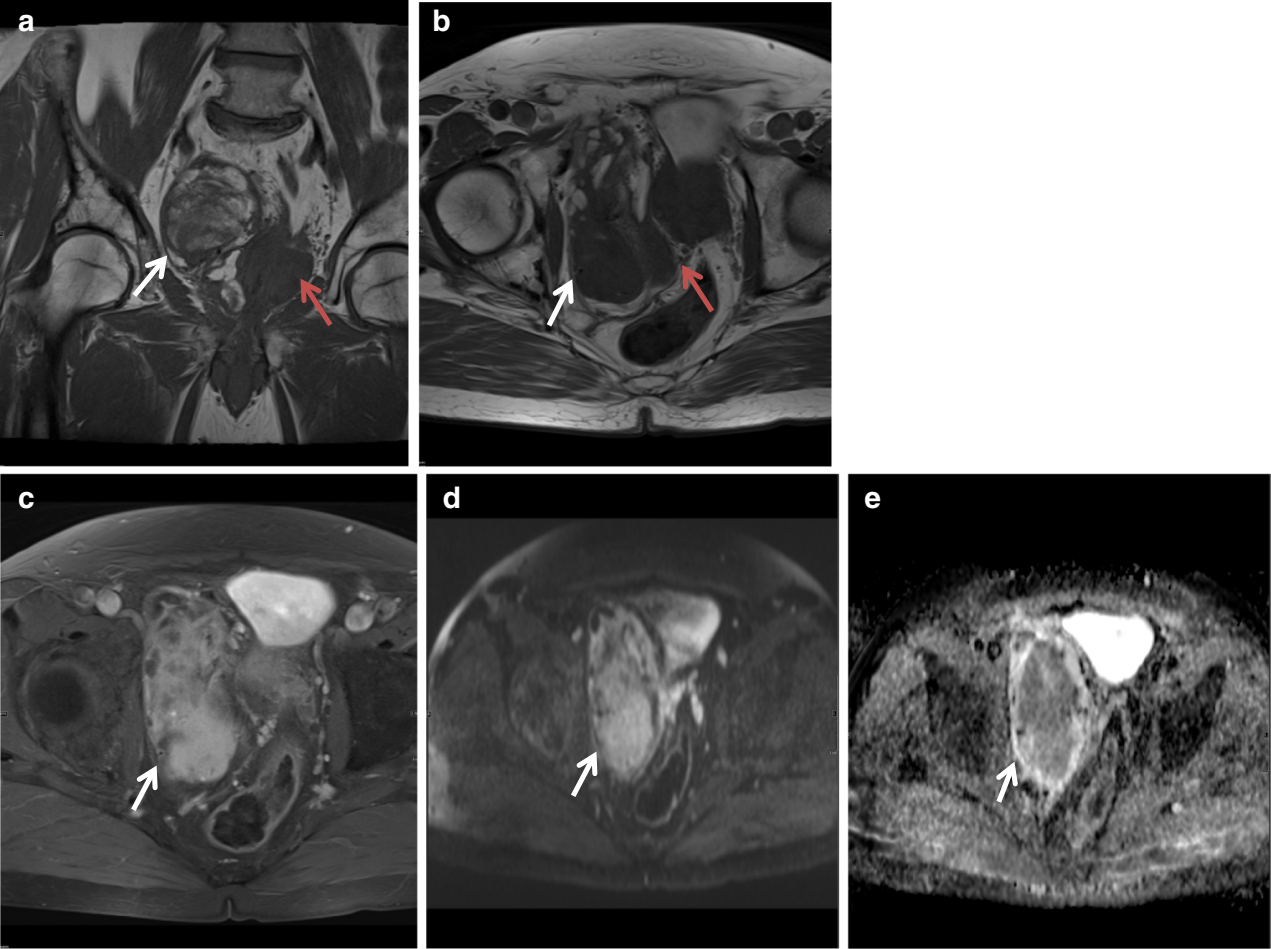
*Liposarcoma* **a** Coronal and (**b**) axial T1-weighted images show a heterogeneous mass (white arrow) which is T1 iso- to hyperintense to prostate (red arrow) arising from the right of the prostate gland which does not fully supress on (**c**) T1 fat saturation with heterogeneous contrast enhancement in the non-fat elements. **d**, **e** DWI and ADC sequences, respectively, show diffusion restriction in the lesion. Surgical resection confirmed liposarcoma

Specialist sarcoma service referral is suggested for any superficial fat containing mass if it is greater than 5 cm or contains a focal solid non-fat component. All deep/intramuscular masses greater than 1 cm are also referred to the sarcoma unit for consideration of biopsy. All intra-abdominal fat containing masses are referred to the sarcoma unit for consideration of biopsy (Figs. 13, 14).

### Parachordoma (also known as myoepithelial carcinoma)

**Fig. 15:**
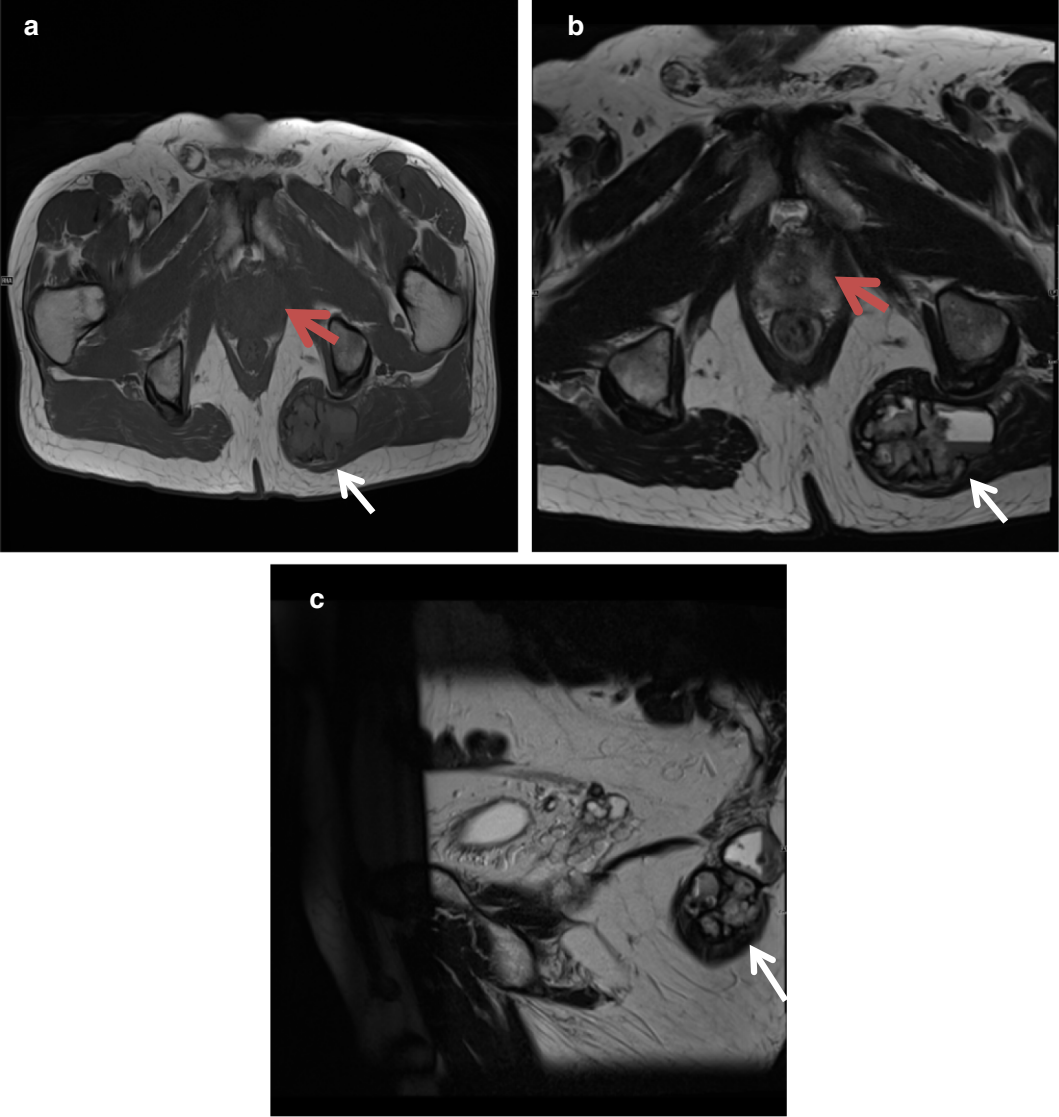
*Parachordoma* **a** Axial T1-weighted, (**b**) axial T2-weighted and (**c**) sagittal T2-weighted images show a lobulated mass within the left gluteus maximus (white arrow). The prostate is labelled by the red arrow. The lesion is iso- to mildly hyperintense to muscle on T1 and hyperintense to muscle on T2 and shows internal fluid–fluid levels in keeping with haemorrhage and cystic change. The lesion did not show diffusion restriction. Surgical resection confirmed myoepithelial carcinoma or parachordoma

Parachordoma is a rare, low-grade malignant musculoskeletal soft tissue tumour which resembles extra-skeletal myxoid chondrosarcoma and chordoma but away from the craniospinal axis [26]. It mainly develops in the deep soft tissue of the distal parts of the limbs, adjacent to deep fascia, tendon, synovium and osseous structures in extremities. It forms a circumscribed firm tumour, with a variety of histological patterns and cytological features, including cords and nests of cells, some of which are vacuolated [26]. Histologically, they show scattered, large, clear, multi-vacuolated cells (physaliferous cells) and on immunohistochemistry, they are positive for cytokeratin and S-100 protein [27]. Differentials include lipomatous tumour and chordoma arising from the sacrum and extending into the gluteal tissues or distant metastasis. Expanded surgical resection is the current mainstay of management. Local recurrence and metastasis from parachordomas have been rarely reported, and as such, ongoing follow-up post-resection is required (Fig. 15).

## Urological

### Bladder wall thickening and hydrocele

In an adequately distended bladder, bladder thickening and trabeculations can be diffuse as in Fig. 2 or focal. Causes of diffuse bladder wall thickening include bladder outlet obstruction, neurogenic bladder and cystitis. On prostate MRI, the most common cause would be chronic bladder outlet obstruction secondary to prostatomegaly. Focal bladder wall thickening is often concerning for malignancy such as urothelial carcinoma or other bladder neoplasms. Rare causes of focal bladder wall thickening include amyloidosis and malakoplakia.

**Fig. 16:**
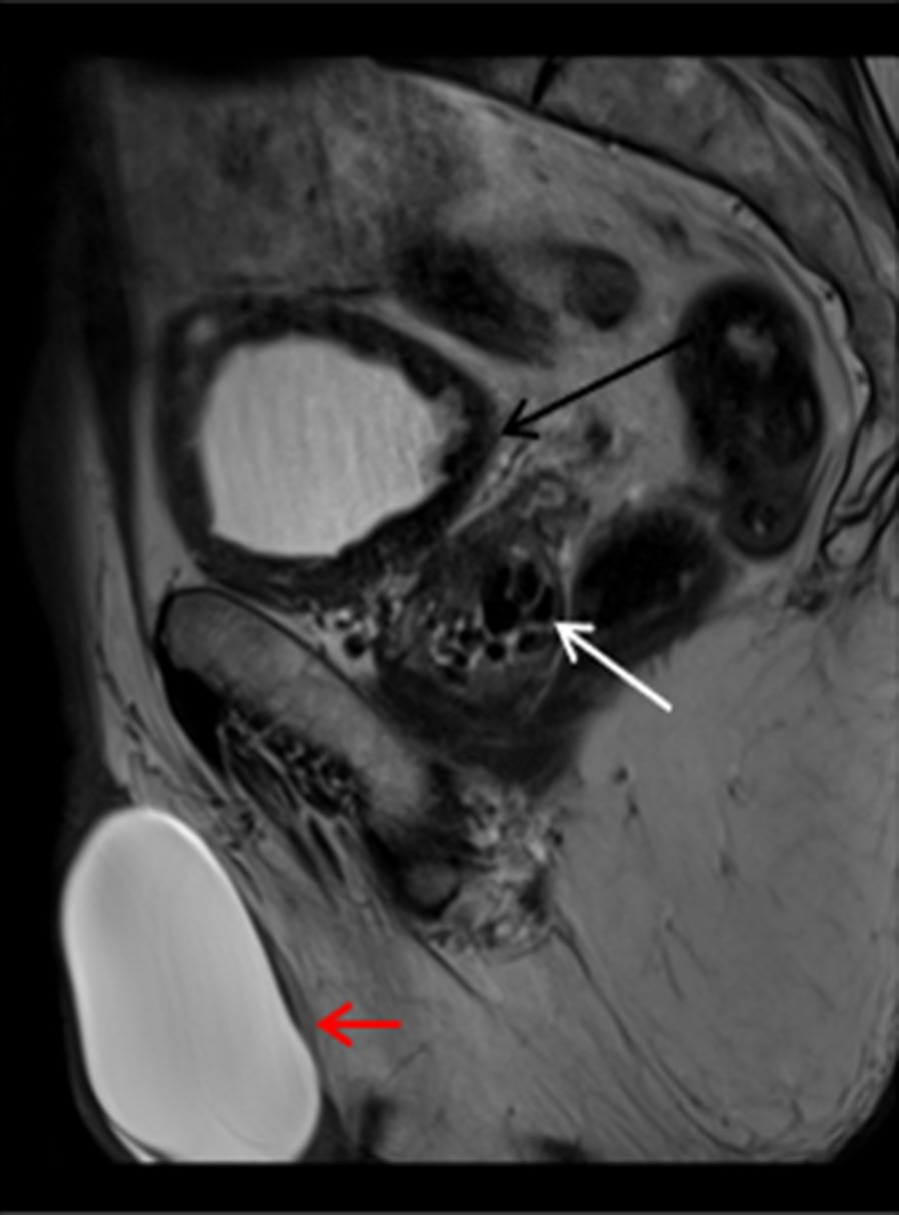
Sagittal T2W image shows large high T2 signal lesion in the right scrotum in keeping with a hydrocele (red arrow), thickened bladder wall which is low T2 signal is in keeping with a trabeculated bladder wall with debris (black arrow). Low T2 signal lesions seen in the prostate (white arrow) represent prostate calcifications as discussed previously in Fig. 2

A hydrocele represents serous fluid within the tunica vaginalis in the scrotum and hence follows fluid characteristics on MRI demonstrating low T1 signal and high T2 signal intensity. If complicated by infection or trauma, they can appear complex with septation or internal debris. Hydroceles, may be acquired or congenital, are common and present with painless enlargement of the scrotum. Congenital hydroceles are more common in children and may be subdivided into communicating or spermatic cord hydrocele [28]. Acquired hydroceles can develop subsequent to trauma, epididymitis, testicular torsion, neoplasm or infarction [28] (Fig. 16).

### Undifferentiated pleomorphic sarcoma (UPS) of the spermatic cord

Primary spermatic cord tumours are rare and clinically significant urologic lesions which may be encountered while examining the prostate on imaging. Table 4 highlights common benign and malignant neoplasms of the spermatic cord that the radiologist should be aware of.

**Table 4:** Spermatic cord neoplasms [30–33]

Benign neoplasms	Fibrolipoma, neurofibroma, paraganglioma, adenomatoid tumour, dermoid cyst, mesothelial cyst, heterotopic ossification, diverticulum, fibrous pseudotumour
Malignant neoplasms	Undifferentiated pleomorphic sarcoma (UPS), lymphoma (primary or secondary), liposarcoma, leiomyosarcoma, rhabdomyosarcoma, osteosarcoma, myxofibrosarcoma, malignant schwannoma, adenocarcinoma, aggressive angiomyxoma

**Fig. 17:**
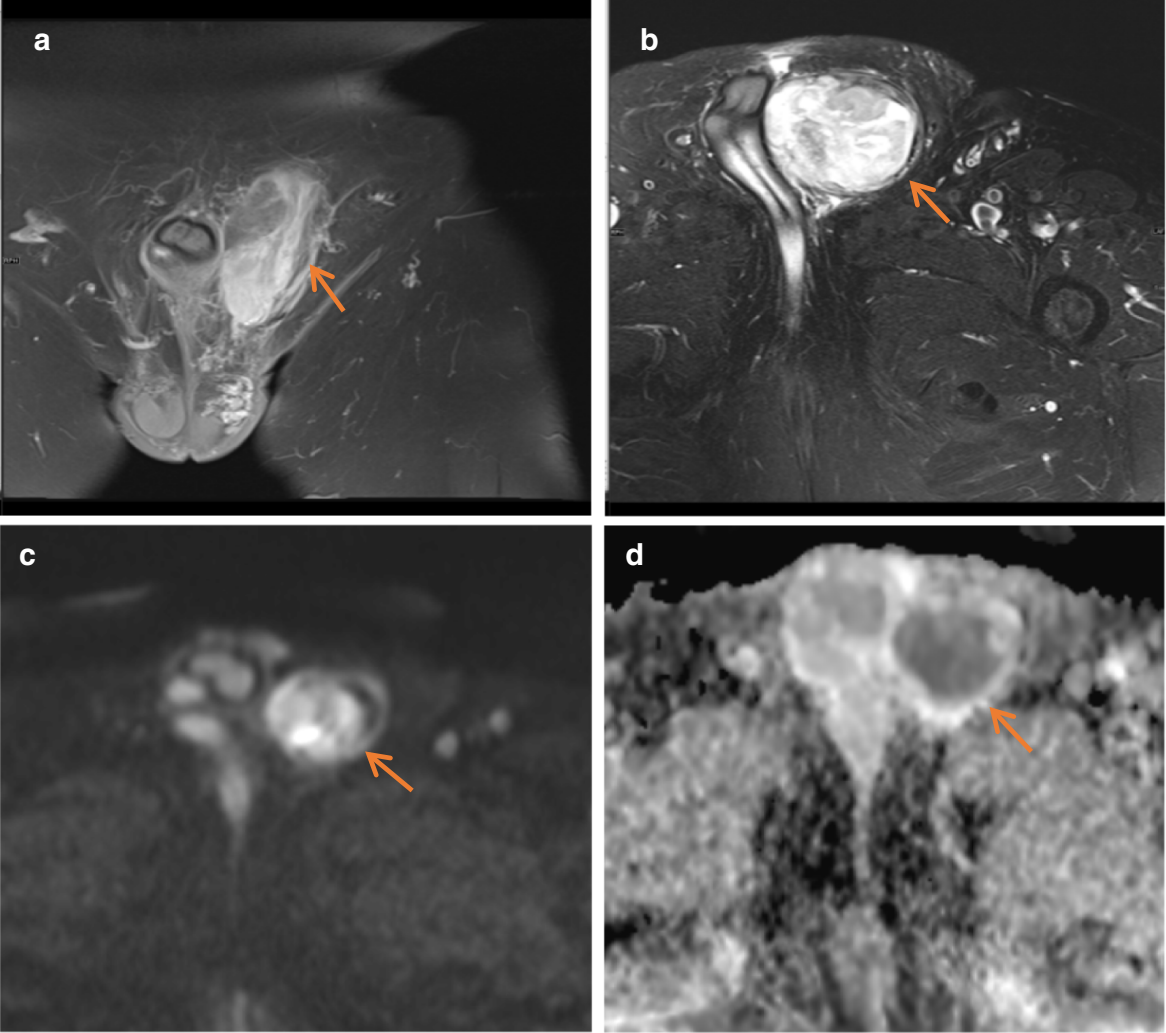
*UPS of the spermatic cord* **a** Coronal T1 fat sat post-contrast and **b** axial T2 fat sat images show a large heterogeneously enhancing high T1 and T2 signal mass within the left inguinal canal and scrotum (red arrow). **c** Axial DWI and **d** ADC images demonstrate a predominantly solid mass with restricted diffusion in keeping with necrosis or cystic change within

Formerly known as malignant fibrous histiocytoma, UPS of the spermatic cord is a rare and aggressive condition commonly presenting in the sixth decade. Patients may present asymptomatic or with a unilateral slow-growing and firm paratesticular mass. There may be secondary hydrocele, pain or symptoms secondary to metastasis [29]. Differentials on clinical examination alone include inguinal hernia, lipoma, hydrocele, haematocele or inflammatory/infective causes such as epididymo-orchitis or tuberculosis. While ultrasound is frequently used for initial investigation and for lesion localisation, many paratesticular lesions can have variable or indeterminate ultrasound appearances and an MRI is ultimately required for further characterisation and depiction of local anatomic relationships [30]. Imaging features of undifferentiated sarcomas are non-specific but on MRI they tend to show heterogeneous post-contrast enhancement with diffusion restriction which may be due to necrosis or haemorrhage. They may also show calcifications inside a solid lesion [31]. Pathologically, the tumour is commonly large with haemorrhage and necrosis [31]. Microscopically, pleomorphic spindle, polygonal and multinucleated giant tumour cells and atypical mitoses, myxoid and inflammatory changes are found [31]. This patient underwent a left orchidectomy and wide local excision and inguinal lymph node dissection which confirmed MFH of the spermatic cord. Given the tendency of sarcomas to infiltrate local tissues and the risk of tumour seeding at operative site, there is a high risk of loco-regional recurrence (Fig. 17).

### Spermatic cord lymphoma

**Fig. 18:**
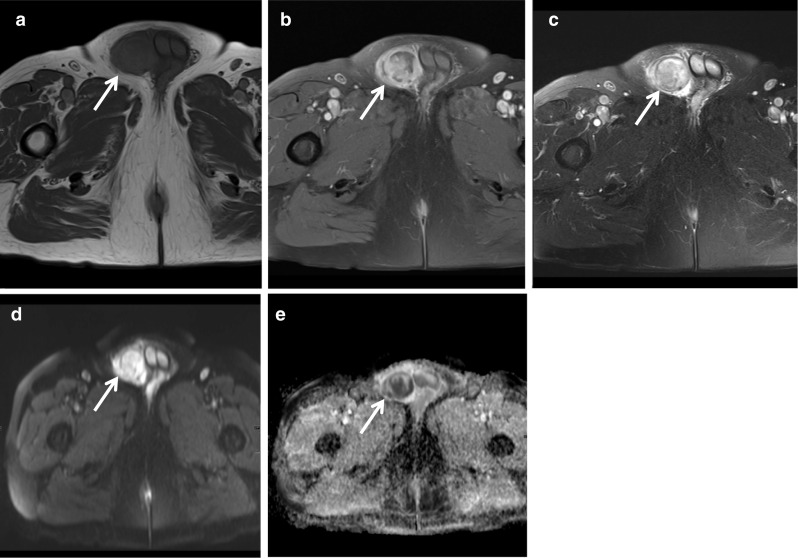
*Spermatic cord lymphoma* **a** Axial T1W image shows a mass within the right inguinal canal (white arrow) which is inseparable from the spermatic cord and is mildly hyperintense to gluteus muscle and shows heterogeneous post-contrast enhancement on (**b**) T1 fat saturation and (**c**) T2 fat saturation sequences. In **d** and **e** axial DWI/ ADC, the right inguinal mass shows restricted diffusion. This was surgically excised and proven to be spermatic cord lymphoma

Spermatic cord lymphoma is an extremely rare condition with poor prognosis. In a literature review published in 2011 by Taguchi et al. [32], only thirty-three cases had been reported. As in our case, they may present with an enlarging scrotal mass alone or if symptomatic may be associated with pain or haematuria. Primary testicular lymphoma is also rare, constituting only 1–7% of all testicular neoplasms and less than 1% of all non-Hodgkin lymphoma [33]. Among all extra-nodal lymphomas, primary lymphoma of the testis and spermatic cord have the worst prognosis with the 5-year overall survival ranging between 70 and 79% [32]. MRI features can be variable but vivid heterogeneous enhancement and diffusion restriction are generally noted. Wide local excision surgery with consideration of neoadjuvant chemotherapy remains the mainstay of treatment albeit with a high risk of recurrence. Discussion at multidisciplinary meetings is highly advised to collaborate on the best treatment pathway (Fig. 18).

### Urothelial carcinoma

Urothelial carcinoma of the ureter accounts for only 1% of all urinary tract malignancies but is the most common primary tumour of the ureter with the greatest incidence in the distal ureter.

**Fig. 19:**
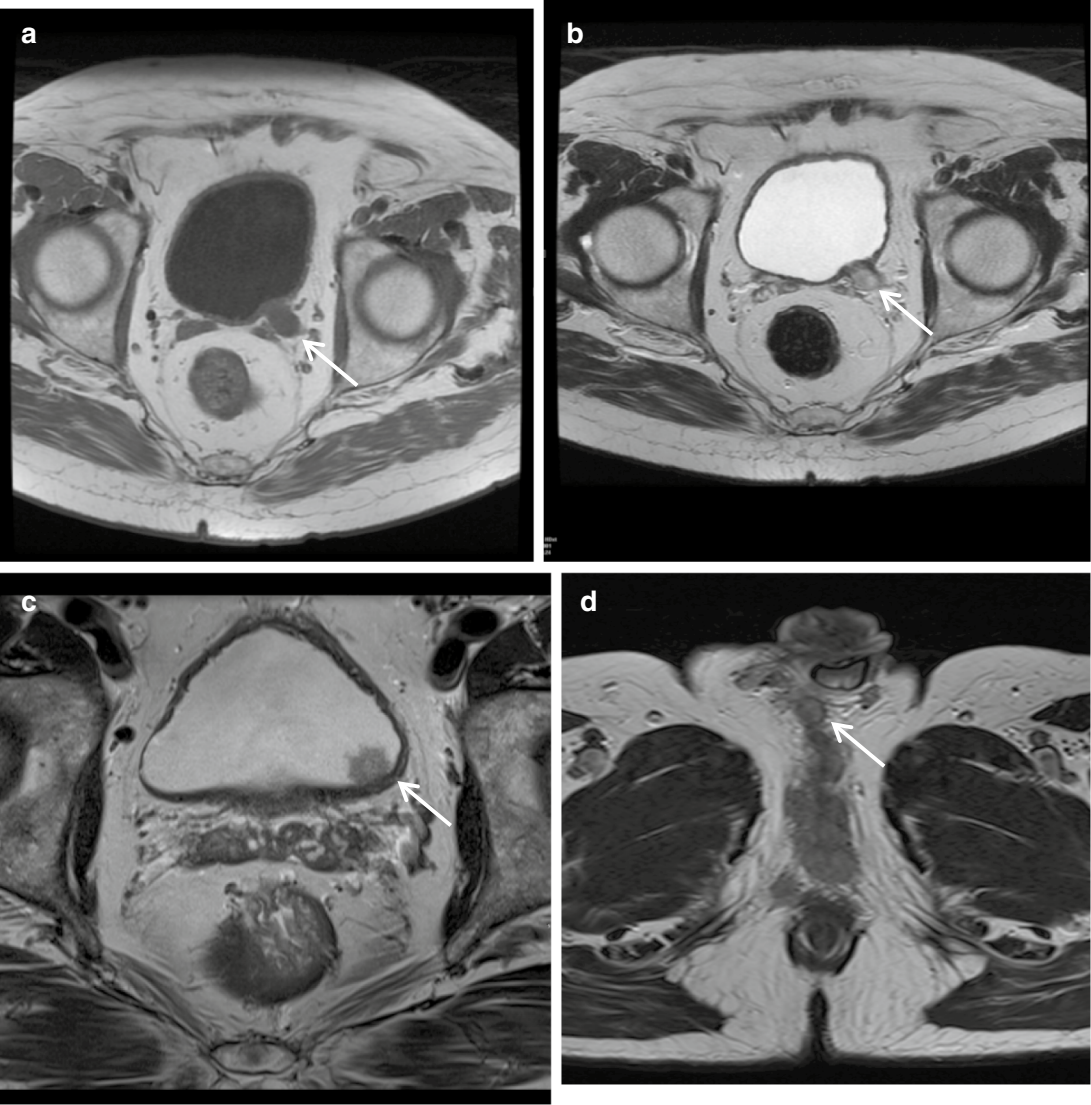
Urothelial carcinoma **a** Axial T1W image shows a slightly T1 hypointense lesion (white arrow) compared to the gluteus maximus muscle in the left vesicoureteric junction. **b** Axial T2W image in the same patient shows the left VUJ filling defect which is T2 hyperintense to bladder wall and gluteal muscle. **c** Is an axial T2W image of a different patient showing an intermediate signal intensity polypoid lesion on the left posterior bladder wall (white arrow). **d** Is an axial T2W image of yet another patient with a history of urothelial carcinoma showing multiple ill-defined, T2 hyperintense to gluteus muscle, nodules in the perineum and extending to the ventral aspect of the penis in keeping with metastases (white arrow)

Urothelial carcinoma is the most common primary neoplasm of the urinary bladder, and bladder urothelial carcinomas are the most common tumour of the entire urinary tract. Bladder carcinomas are 50 times more common than urothelial carcinomas of the renal pelvis and 100 times more common than of the ureter [34]. It has a male predilection (4:1) and the average age of presentation is 65 years [34]. On MRI, they are usually T1 isointense to muscle and slightly T2 hyperintense to muscle and show post-contrast enhancement. DWI is increasingly used to diagnose and localise urothelial carcinoma. At DWI with high b values of 800-1000 s/mm2, bladder cancers generally show a homogeneous hyperintense signal, which reflects homogeneous tissue composition [35]. The sensitivity, specificity and accuracy of DWI for detecting bladder cancer were reported to be 91–100%, 77–91% and 81–96%, respectively [35] (Fig. 19).

## Colorectal

### Inguinal hernia

Inguinal hernia is the commonest type of abdominal wall hernia (up to 80%). They are most often acquired and have a male predominance with M:F ratio of upto 7:1 [36]. Table 5 lists some other differentials for a mass in the inguinal canal.

**Table 5:** Differentials for inguinal canal mass that may be seen on prostate MRI [37, 38]

Benign	Inguinal hernia-(fat or bowel containing, direct or indirect), mesh or plugs, ascites in tunica vaginalis, lipoma, aneurysm, arteriovenous fistula, abscess, haematoma
Malignant	Lymph node metastases (most frequently from vagina, vulva, penis, lower part of the rectum, anus and lower extremities)

Inguinal hernias may be direct or indirect, with indirect being five times more common. Direct hernias by contrast often descend through a weakened defect in the abdominal wall transversalis fascia within the Hesselbach triangle which is bordered by the rectus abdominis muscle medially, inferior epigastric vessels superolaterally and the inguinal ligament inferiorly. Hence, direct inguinal hernias occur inferomedial to the inferior epigastric vessels. Indirect hernias are more common, pass through the deep inguinal ring, usually anterior to the spermatic cord and lateral to the inferior epigastric vessels and Hesselbach triangle.

In men, the contents of the hernia can extend through the superficial ring into the scrotum, while in women, they can follow the round ligament into the labia majora [37]. Hernias may contain intra-abdominal fat or bowel, either small bowel or large bowel or rarely reproductive organs. When containing bowel the hernias may become obstructed in which case the bowel loops will become distended with surrounding fat stranding and fluid. Although diagnosis is usually made based on clinical examination, ultrasound and/ or CT, MRI can help identify occult inguinal hernia, although this is not standard practice given the associated increased and often unnecessary costs [36].

**Fig. 20:**
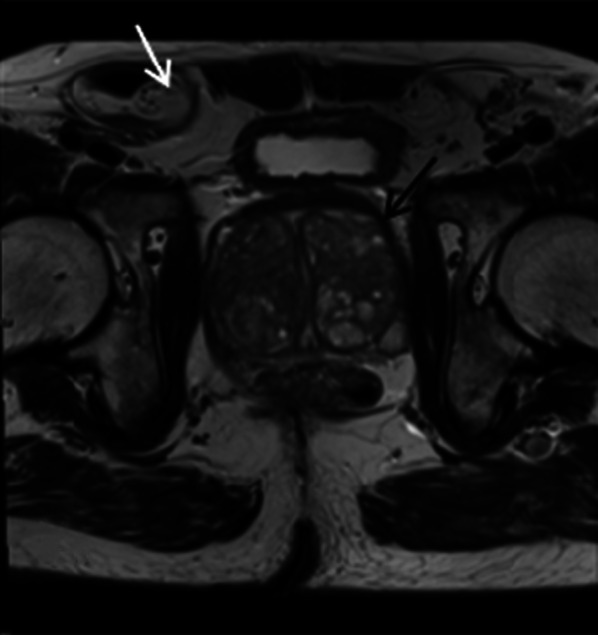
Axial T2W images demonstrate a moderately high T2 signal mass in the right inguinal region (white arrow) which was contiguous with small bowel on serial images and in keeping with a right-sided small bowel containing inguinal hernia. The prostate is labeled by the black arrow

Occasionally evidence of prior mesh repair or plugs may be seen. A mesh often appears as low signal on T1/T2 with adjacent susceptibility artefact. Plugs often appear as rounded masses at the opening of the internal ring, and they often appear low signal on T1 and T2 but depending on surrounding inflammatory response and timing of the operation may have mixed signal intensities (Fig. 20).

### Ascites

Ascites refers to the pathologic accumulation of fluid within the peritoneal cavity. There may be 50-75 ml physiological fluid in this cavity which can be referred to simply as free fluid. Ascites may be of transudative or exudative nature and both have different aetiologies and imaging features [39]. Similar to simple fluid elsewhere, on MRI, transudative ascites is low signal on T1 and high signal on T2 [39].

**Fig. 21:**
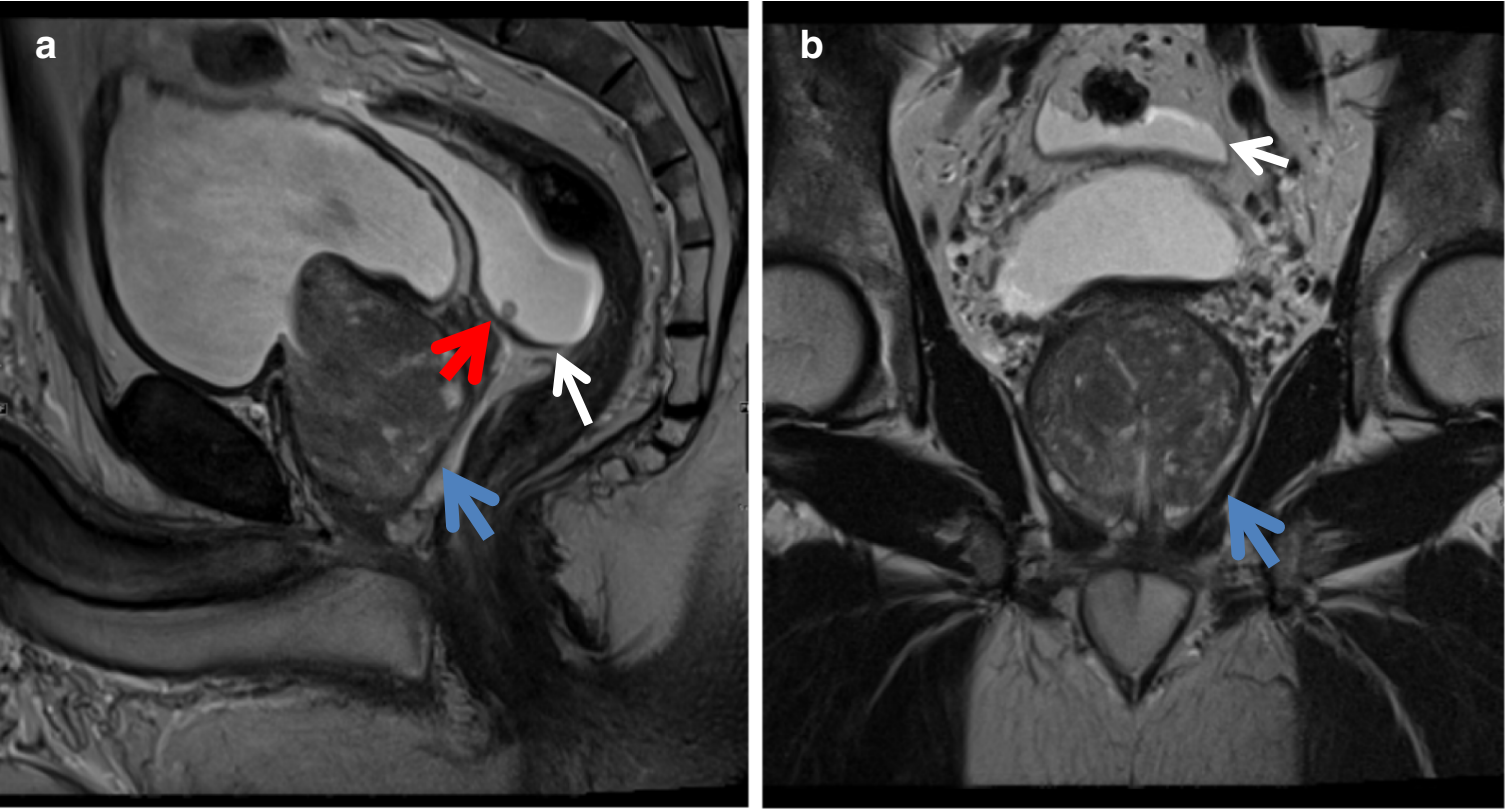
*Ascites* **a** Sagittal and **b** coronal T2-weighted images demonstrate high T2 signal free fluid (white arrows) of similar intensity to the urinary bladder in keeping with ascites. The fluid is seen posterior and superior to the prostate (blue arrow) at the base of the bladder. **a** Also shows a 4 mm nodule (red arrow) in the anterior inferior aspect of the free fluid

The other type of ascites is exudative ascites which is characterised by high protein and high specific gravity and can be secondary to infection, ischaemia, peritoneal carcinomatosis, peritonitis or pancreatitis [40]. On imaging, owing to their complex nature, they are mildly hyperdense on CT compared to transudative ascites with between fifteen to thirty hounsfield units and on MRI, can result in intermediate T1 and T2 signal due to internal complexity and protein [39, 41]. Peritoneal thickening, enhancement, loculation, septation and nodularity may be seen in exudative ascites. If nodular thickening is present, as seen in Fig. 19, this can be seen in malignant ascites and if an underlying malignancy is not known, the patient should be further investigated to exclude this as a cause (Fig. 21).

### Rectal Gastrointestinal Stromal Tumours (GIST)

**Fig. 22:**
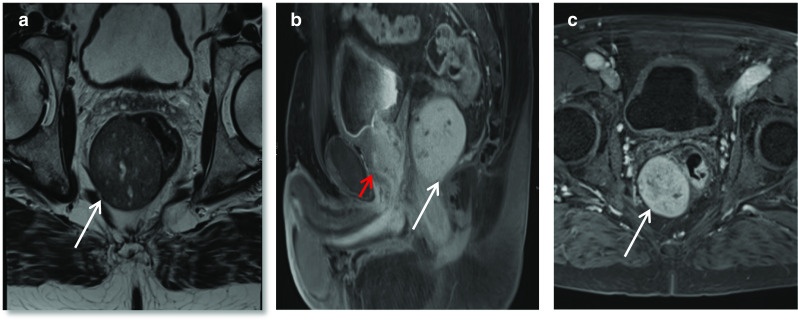
*GIST* Well-circumscribed submucosal 5 cm heterogeneous mass (white arrow) arising from the right posterolateral wall of the lower rectum is (**a**) predominantly T2 hyperintense relative to rectal wall with (**b**) and (**c**) sagittal and axial T1 fat saturation post-contrast sequences showing vivid contrast enhancement. The mass is seen posterior to the prostate in B (red arrow). Biopsy confirmed spindle-type histologically low-grade GIST

Gastrointestinal stromal tumours are the most common mesenchymal tumours of the gastrointestinal tract accounting for approximately 5% of all sarcomas. The stomach and small bowel are the most commonly involved sites with GIST of the anal canal and rectum only accounting for about 5% of all GISTs in the gastrointestinal tract [42]. They are typically submucosal tumours with usually intact mucosa on pathological and imaging assessment. They are thought to arise from the interstitial cells of Cajal with 95% staining positive for CD 117 9c-KIT and 70% for CD34 [42]. On histology they are a relatively cellular tumour arising from the muscularis propria composed of spindle cells (70–80%) and plump epithelioid cells (20–30%) [42]. They can have variable imaging appearances depending on the presence of necrosis, haemorrhage or cystic change but generally demonstrate a low T1 signal intensity solid component and high T2 signal intensity solid component with post-contrast enhancement. Surgical resection is the mainstay of treatment. The Choi response criteria for GIST, which proposed that tumour attenuation could provide an additional measure of response to imatinib therapy (adjuvant chemotherapy), is used to assess the treatment [43] (Fig. 22).

### Rectal villous adenomas

**Fig. 23:**
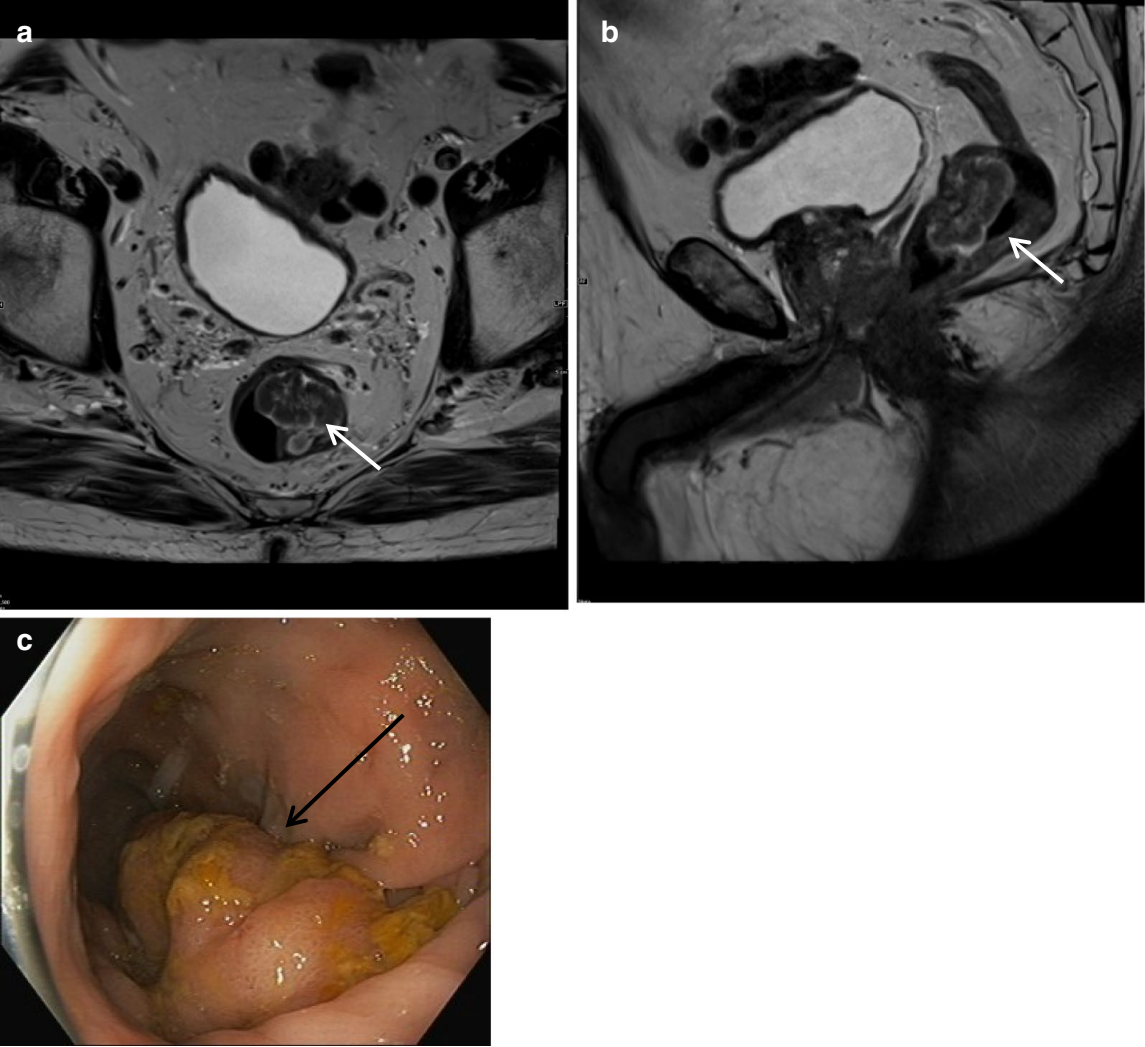
*Rectal villous adenoma* **a** Axial and (**b**) sagittal T2W images show a large lobulated polypoid mass within the lower rectum at 2 cm from anal verge (white arrow). The mass shows thick T2 hyperintense layer along the surface with heterogeneous intermediate to high-signal intensity within the lesion. **c** Colonoscopy and polypectomy confirmed villous/ tubulovillous adenoma (black arrow) with low-grade dysplasia

Villous lesions are advanced adenomas with malignant potential. Villous adenomas represent less than 5% of all adenomas [43]. Although patients with colorectal villous polyps can be asymptomatic, they may present with gastrointestinal symptoms such as constipation or bowel obstruction, acute PR bleeding or chronic iron deficiency anaemia. On MRI, they tend to have low T1 signal intensity with high T2 signal peripherally and can be heterogeneous often in a polypoid morphology. Given the difficulty in diagnosing high-grade dysplasia or invasive adenocarcinoma on biopsy alone, they are usually excised as in the below case. In our experience, rectal polyps are detected as incidental findings on prostate MRI less frequently than prostate cancer is detected incidentally on rectal MRI; however, the rectum remains an important check area (Fig. 23).

### Rectal adenocarcinoma

**Fig. 24:**
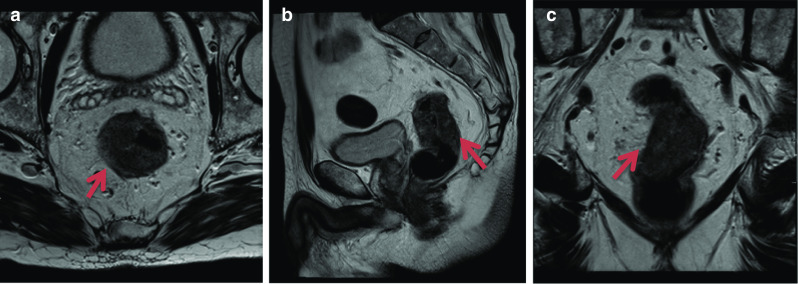
*Rectal adenocarcinoma* Axial, sagittal and coronal T2-weighted images showing a semi-annular mass (red arrow), posterior to the prostate and within the rectum which is T2 hyperintense to gluteal muscle and extending from 3 to 1 o’clock causing significant luminal narrowing. Colonoscopy and biopsy proved rectal adenocarcinoma

Can be incidentally seen on prostate MRI. The reported frequency of synchronous prostate and rectal cancer is low with some studies reporting an incidence of 0.1–0.3%. It is important to review the rectum as most prostate and rectal MRI protocols share the same high-resolution T2 and DWI sequences. Mesorectal nodal metastases from prostate cancer are increasingly being recognised with PSMA PET; however, it is prudent that if abnormal nodes are seen in the mesorectum careful inspection of the rectum should be made [44] (Fig. 24).

## Vascular

### Periprostatic venous varix

**Fig. 25:**
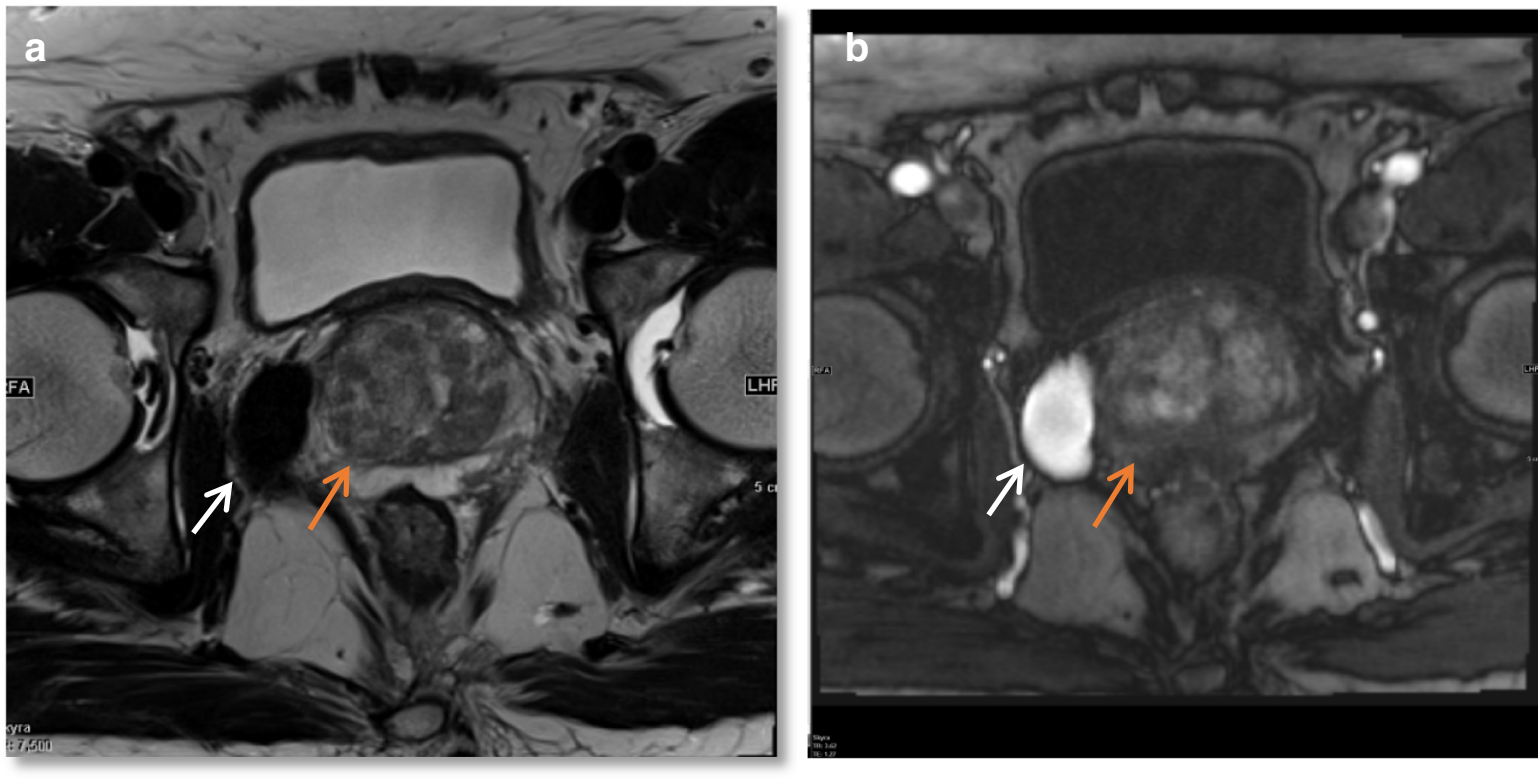
*Periprostatic venous varix* **a** Axial T2W image shows a flow void (white arrow) to the right of the prostate gland (red arrow) and (**b**) dynamic post-contrast axial T1W image shows dynamic enhancement confirming a right-sided periprostatic venous varix

The prostatic venous plexus is a network of veins around the anterolateral aspect of the prostate and anterior to the bladder. The tributaries include deep dorsal vein of the penis, anterior vesical rami and prostatic rami. The prostatic plexus then drains into the vesical and internal iliac veins [14]. It can be identified correctly based on the location coursing along the lateral margin of the peripheral zone, tubular morphology when tracked on consecutive slices and assessing the contralateral vessels for symmetry [16]. Identification of the varix is useful preoperatively for planning because their inadvertent disruption can lead to haemorrhage. They normally show flow voids on T1- and T2-weighted sequences and enhance on post-contrast sequences. When thrombosed, they can be identified due to loss of normal flow void on MR (Fig. 25).

### Lymphadenopathy

Identification or exclusion of abnormal lymph nodes is an important component of tumour staging and also in assessing the extent and prognosis of the underlying disease [39]. On a prostate MRI, lymphadenopathy may be incidentally detected and in the absence of a primary tumour in the prostate, this warrants a search for the primary with close scrutiny of the bladder and rectum on the MRI as well as consideration of another systemic pathology. MRI is limited in distinguishing between benign and malignant lymph node enlargement due to lymphatic tissue and tumour having similar T1 and T2 relaxation times and proton densities [45]. Both CT and MRI largely rely on size to indicate nodal metastasis with size generally greater than 10 mm in short axis considered suspicious. Lymph nodes that are not enlarged by metastasis have a mean diameter of only a few millimetres and are usually not visualised on MR images [45]. In the pelvic region, lymph nodes may be hard to differentiate from elongated iliac vessels especially on T1W images which depict both lymph nodes and vessels with low-signal intensity and PDw images may be useful which depict vessels as flow voids [45]. More reliable features which can be seen in advanced nodal metastasis include diffusion restriction, central necrosis (seen as a hyperintensity within a metastatic node on T2W images), lymph node conglomeration and multiple enlarged features [45].

**Fig. 26:**
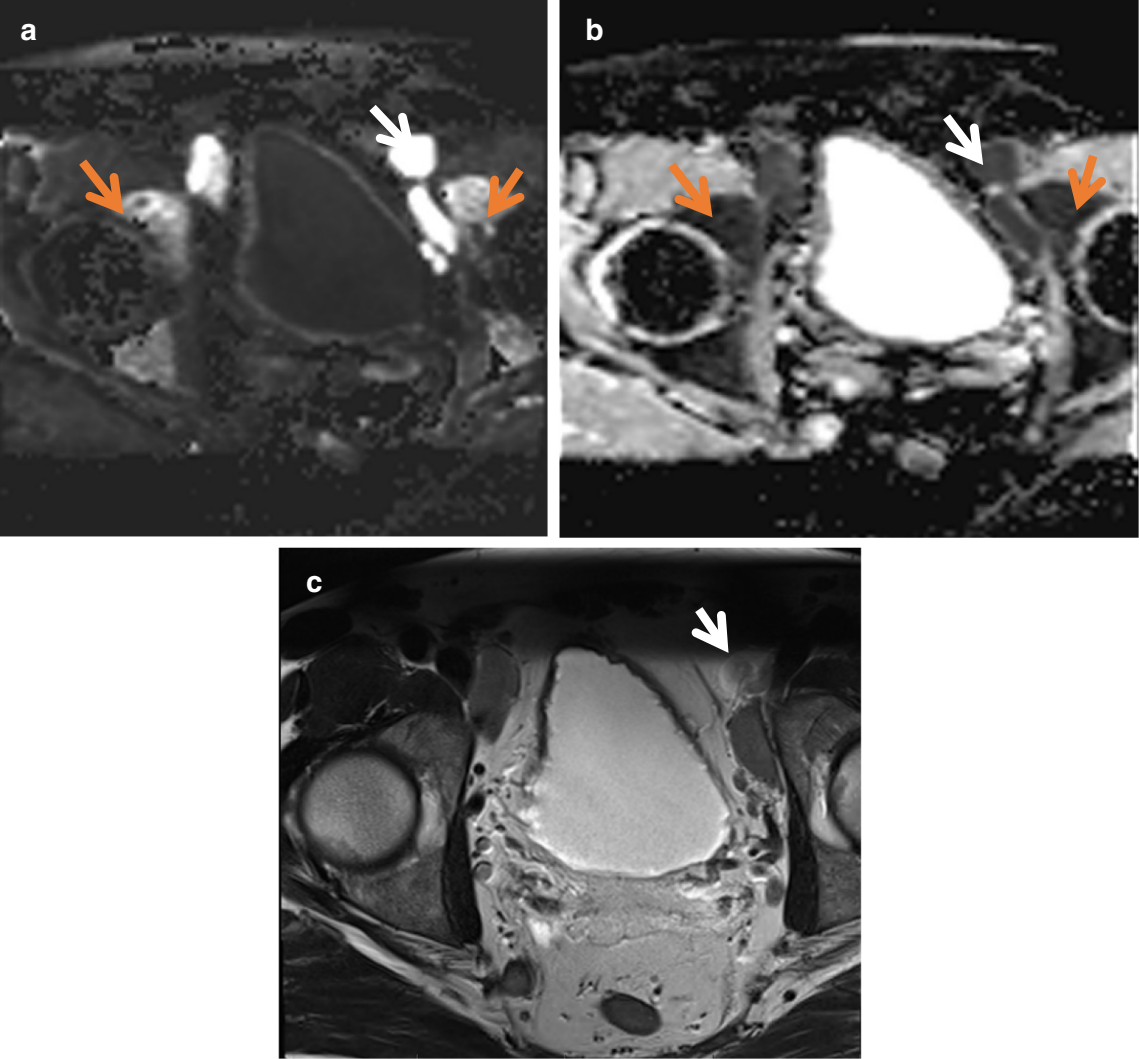
*Lymphadenopathy in a patient with multiple myeloma* **a** Axial DWI and (**b**) ADC images showing diffusion restriction in a rounded lesion to the left of the urinary bladder anteriorly (white arrows) in keeping with nodal metastasis in left external iliac lymph nodes. Diffusion restriction also in the acetabulum bilaterally (red arrows) in keeping with marrow infiltration. **c** Axial T2W images demonstrate the left external iliac lymph node is iso- to hyperintense relative to surrounding fat which is seen in nodal metastasis

Abnormal lymph nodes may be associated with primary prostatic-related pathology or other systemic pathology and may be incidentally noted when reporting prostate MRI. If prior clinical history of haematological malignancy has been provided, such as in the case of Fig. 24, where there was a background of multiple myeloma, this can be extremely useful to be aware of when reporting and avoid over-investigation if this is known and stable on comparison imaging. While marrow infiltration is best seen on T1 as low signal lesion causing loss of normal marrow signal, on T2W imaging it is seen as high signal compared to surrounding marrow. Again, as differentials for this are broad, including trauma, infection or spondyloarthropathies, clinical background and comparison imaging, correlation with CT or plain film if available, is of immense value in determining both benignity and chronicity of these abnormalities (Fig. 26).

## Conclusion

Prostate MRI continues to expand in its utility in the diagnosis, management and surveillance of prostate cancer. It allows detection of both clinically significant and indolent incidental findings in the field of view including abdominal and pelvic organs, vessel and bones. There are a myriad of lesions which can either arise from or be in the vicinity of the prostate that can be missed or incorrectly diagnosed by the radiologist not familiar with reporting prostate MRI commonly.

A systemic approach with review of the distal ureters, bladder and rectum as check areas, is important as several incidental findings in these locations can be neoplastic. Sound knowledge of the anatomical structures in the field of view will help guide towards the origin of the lesion and assist in characterising as some locations are characteristic such as the prostatic venous plexus around the posterolateral aspect of the prostate gland. Localising the pathology to an organ significantly helps in narrowing the differential list. Some MRI signal characteristics of haemorrhage and calcification are important to recognise as they provide important clues for the differential diagnosis. Being aware of the clinical background such as recent intervention or haematuria in the case of haemorrhage, or history of intravesical BCG for TCC in the case of a prostatic abscess, can greatly contribute to increased confidence in making a diagnosis. Often leiomyomas and benign mesenchymal tumours can be difficult to differentiate based on imaging alone and a biopsy for histological and immunohistochemistry may be inevitable to confirm the pathology.

When identifying a cystic structure, the main considerations should be between a utricle cyst and a mullerian duct cyst. The former is typically smaller, midline and does not extend above the base of the prostate. When identifying a solid lesion assess for the presence of blood products or fat as these can narrow the differential. Once the presence of a solid mass separate to the prostate is established, it can be challenging to differentiate benign and malignant soft tissue tumours and often a targeted biopsy is required. However, being able to recognise common lesion characteristics, in conjunction with patient demographics and background, helps reach a succinct and accurate list of differential diagnoses.

It is important for the reporting radiologist to be aware that these exist to be able to make an adequate assessment and also that although they may be benign or malignant, their detection and follow-up often has implications on clinical management, patient anxiety and increased cost. This is especially important given that prostate MRI is often performed for patients that have known or strong suspicion and risk for prostate cancer and is already a cohort with significant stressors and anxiety. Hence, it is prudent that radiologists are able to develop skills in diagnosing these lesions and collaborating with the referrer on a management plan.

## Data Availability

Not applicable.

## Abbreviations

ADC: Apparent diffusion coefficient
ALT: Atypical lipomatous tumour
BCG: Bacillus calmette–guérin
BPH: Benign prostatic hyperplasia
CT: Computed tomography
DWI: Diffusion-weighted imaging
FDG-PET: Fluorodeoxyglucose-positron emission tomography
GIST: Gastrointestinal stromal tumour
MFH: Malignant fibrous histiocytoma
mpMRI: Multi-parametric magnetic resonance imaging
MRI: Magnetic resonance imaging
NVB: Neurovascular bundle
PET: Positron emission tomography
PIRADS: Prostate imaging reporting and data system
PSA: Prostate-specific antigen
SFT: Solitary fibrous tumour
SMA: Smooth muscle actin
STUMP: Smooth muscle tumours of uncertain malignant potential
TRUS: Transrectal ultrasound
UPS: Undifferentiated pleomorphic sarcoma
VUJ: Vesicoureteric junction

## References

[1] Sarkar S, Das S (2016). A review of imaging methods for prostate cancer detection. Biomed Eng Comput Biol.

[2] Razek AA, Elhanbly S, Eldeak A (2010). Transrectal ultrasound in patients with hematospermia. J Ultrasound.

[3] Neto JA, Parente DB (2013). Mulitparametric magnetic resonance imaging of the prostate. Magn Reson Imaging Clin N Am.

[4] American College of Radiology. Prostate Imaging—Reporting and Data System. 2019. Version 2.1. PI-RADS

[5] Shin T, Smyth TB, Ukimura O (2018). Diagnostic accuracy of a five-point Likert scoring system for magnetic resonance imaging (MRI) evaluated according to results of MRI/ultrasonography image-fusion targeted biopsy of the prostate. BJU Int.

[6] Sklinda K, Frączek M, Mruk B, Walecki J (2019) Normal 3T MR anatomy of the prostate gland and surrounding structures. Adv Med 2019, Article ID 3040859, 9 pages. 10.1155/2019/3040859 10.1155/2019/3040859 PMC6558623 31276002

[7] Sherrer RL, Lai WS, Thomas JV, Nix JW, Rais-Bahrami S (2017). Incidental findings on multiparametric MRI performed for evaluation of prostate cancer. Abdom Radiol (NY).

[8] Cutaia G, Tosto G, Cannella R (2020). Prevalence and clinical significance of incidental findings on multiparametric prostate MRI. Radiol Med.

[9] Zaidi S, Gandhi J, Seyam O (2018). Etiology, diagnosis, and management of seminal vesicle stones. Curr Urol.

[10] Panebianco V, Barchetti F, Barentsz J (2015). Pitfalls in interpreting mp-MRI of the prostate: a pictorial review with pathologic correlation. Insights Imaging.

[11] Nghiem HT, Kellman GM, Sandberg SA (1990). Cystic lesions of the prostate. Radiographics.

[12] Curran S, Akin O, Agildere AM (2007). Endorectal MRI of prostatic and periprostatic cystic lesions and their mimics. AJR Am J Roentgenol.

[13] Miao C, Liu S, Zhao K, Zhu J, Tian Y, Wang Y (2019). Treatment of Mullerian duct cyst by combination of transurethral resection and seminal vesiculoscopy: an initial experience. Exp Therapeutic Med.

[14] Susan Standring. Gray's Anatomy. (2015) ISBN: 9780702052309

[15] Asbach HW, Melekos M (1982). Cowper's gland duct cyst. Int Urol Nephrol.

[16] Kitzing YX (2016). Benign conditions that mimic prostate carcinoma: MR Imaging features with histopathologic correlation. Radiographics.

[17] Eom JH, Yoon JH, Lee SW, Kim HS, Park TY, Bang CS (2016). Tuberculous prostatic abscess with prostatorectal fistula after intravesical bacillus Calmette-Guérin immunotherapy. Clin Endosc.

[18] Chalhoub K, Abou Zahr R, Mansour E, Aoun M, Jabbour M (2019). Primary mature cystic teratoma compressing the prostate in a 28-year-old male: a case report and literature review. Case Rep Urol.

[19] Lim DJ, Hayden RT, Murad T, Nemcek AA, Dalton DP (1993). Multilocular prostatic cystadenoma presenting as a large complex pelvic cystic mass. J Urol.

[20] Albert PS, Sinatra T, Nagamatsu GR (1974). Retroperitoneal leiomyoma presenting as prostatic mass. Urology.

[21] Vergauwen O, Vereecke E, Villeirs G (2018). Prostatic leiomyoma—multiparametric prostate MRI features. J Belg Soc Radiol.

[22] Venyo AK-G (2015) A review of the literature on primary leiomyosarcoma of the prostate gland. Adv Urol 2015, Article ID 485786, 15 pages (2015) 10.1155/2015/485786 PMC4659954 26640482

[23] Moureau-Zabotto L (2012). Solitary fibrous tumor of the prostate: case report and review of the literature. Case Rep Oncol.

[24] Kransdorf MJ, Bancroft LW, Peterson JJ, Murphey MD, Foster WC, Temple HT (2002). Imaging of fatty tumors: distinction of lipoma and well-differentiated liposarcoma. Radiology.

[25] Nardo L, Abdelhafez YG, Acquafredda F (2020). Qualitative evaluation of MRI features of lipoma and atypical lipomatous tumor: results from a multicenter study. Skeletal Radiol.

[26] Zhang J, Wang H, Cheng X, Wang M, Zhu Y (2013). A case of parachordoma on the chest wall and literature review. J Can Res Ther.

[27] Folpe AL, Agoff SN, Willis J, Weiss SW (1999). Parachordoma is immunohistochemically and cytogenetically distinct from axial chordoma and extraskeletal myxoid chondrosarcoma. Am J Surg Pathol.

[28] Abbas TO, Abdelkareem M, Alhadi A, Kini V, Chandra P, Al-Ansari A, Ali M (2018). Suspected testicular torsion in children: diagnostic dilemma and recommendation for a lower threshold for initiation of surgical exploration. Res Rep Urol.

[29] Nicola R, Menias CO, Dahiya N (2017). Review of paratesticular pathology: findings on ultrasound and MRI. Abdom Radiol (NY).

[30] Woodward PJ, Schwab CM, Sesterhenn IA (2003). Extratesticular scrotal masses: radiologic-pathologic correlation. Radiographics.

[31] Dagur G, Gandhi J, Kapadia K (2017). Neoplastic diseases of the spermatic cord: an overview of pathological features, evaluation, and management. Transl Androl Urol.

[32] Taguchi S, Takahashi S, Iida K, Mizutani T, Yamaguchi K, Tominaga T, Niwa N, Yoshimi M, Takahashi T, Homma Y (2012) Spermatic cord lymphoma: a case report and literature review. Case Rep Med 2012, Article ID 513707, 4 pages. 10.1155/2012/513707 10.1155/2012/513707 PMC3295330 22431934

[33] Şentürk AB, Ekici M, Ersoy H (2015). Primary Testicular B-cell Lymphoma. J Urol Surg.

[34] Leder RA, Dunnick NR (1990). Transitional cell carcinoma of the pelvicalices and ureter. AJR Am J Roentgenol.

[35] Yoshida S, Takahara T, Kwee TC, Waseda Y, Kobayashi S (2017). Fujii Y (2017) DWI as an imaging biomarker for bladder cancer. AJR Am J Roentgenol.

[36] Miller J, Cho J, Michael MJ, Saouaf R, Towfigh S (2014). Role of imaging in the diagnosis of occult hernias. JAMA Surg.

[37] Bhosale PR, Patnana M, Viswanathan C (2008). The inguinal canal: anatomy and imaging features of common and uncommon masses. Radiographics.

[38] Burkhardt JH, Arshanskiy Y, Munson JL, Scholz FJ (2011). Diagnosis of inguinal region hernias with axial CT: the lateral crescent sign and other key findings. Radiographics.

[39] Thoeni RF (1995). The role of imaging in patients with ascites. AJR Am J Roentgenol.

[40] Jeffery J (2001). Ascitic fluid analysis: the role of biochemistry and haematology. Hosp Med.

[41] Jolles H, Coulam CM (1980). CT of ascites: differential diagnosis. AJR Am J Roentgenol.

[42] King DM (2005). The radiology of gastrointestinal stromal tumours (GIST). Cancer Imaging.

[43] Lubner MG, Menias CO, Johnson RJ, Gaballah AH, Shaaban A, Elsayes KM (2018). Villous gastrointestinal tumors: multimodality imaging with histopathologic correlation. Radiographics.

[44] Sturludóttir M, Martling A, Carlsson S, Blomqvist L (2015). Synchronous rectal and prostate cancer–the impact of MRI on incidence and imaging findings. Eur J Radiol.

[45] Taupitz M (2007) Imaging of Lymph nodes—MRI and CT. In: Hamm B, Forstner R (eds) MRI and CT of the female pelvis. Medical radiology (diagnostic imaging). Springer, Berlin. 10.1007/978-3-540-68212-7_15

